# EphrinB/EphB Signaling Controls Embryonic Germ Layer Separation by Contact-Induced Cell Detachment

**DOI:** 10.1371/journal.pbio.1000597

**Published:** 2011-03-01

**Authors:** Nazanin Rohani, Laura Canty, Olivia Luu, François Fagotto, Rudolf Winklbauer

**Affiliations:** 1 Department of Biology, McGill University, Montreal, Quebec, Canada; 2 Department of Cell and Systems Biology, University of Toronto, Toronto, Canada; Osaka University, Japan

## Abstract

**Background:**

The primordial organization of the metazoan body is achieved during gastrulation by the establishment of the germ layers. Adhesion differences between ectoderm, mesoderm, and endoderm cells could in principle be sufficient to maintain germ layer integrity and prevent intermixing. However, in organisms as diverse as fly, fish, or amphibian, the ectoderm-mesoderm boundary not only keeps these germ layers separated, but the ectoderm also serves as substratum for mesoderm migration, and the boundary must be compatible with repeated cell attachment and detachment.

**Principal Findings:**

We show that localized detachment resulting from contact-induced signals at the boundary is at the core of ectoderm-mesoderm segregation. Cells alternate between adhesion and detachment, and detachment requires ephrinB/EphB signaling. Multiple ephrinB ligands and EphB receptors are expressed on each side of the boundary, and tissue separation depends on forward signaling across the boundary in both directions, involving partially redundant ligands and receptors and activation of Rac and RhoA.

**Conclusion:**

This mechanism differs from a simple differential adhesion process of germ layer formation. Instead, it involves localized responses to signals exchanged at the tissue boundary and an attachment/detachment cycle which allows for cell migration across a cellular substratum.

## Abstract 2

Tissue boundary formation in the early vertebrate embryo involves cycles of cell attachment and detachment at the boundary, and cell contact-dependent signaling by membrane-bound EphB receptors and ephrinB ligands.

## Author Summary

The formation and maintenance of tissue boundaries is an essential feature of multicellular animals, including humans. Using the frog embryo as a model system, we describe a mechanism of tissue separation that involves repeated cycles of cell attachment and detachment at the boundary between two adjacent tissues. Molecularly, this mechanism is based on a signal exchange across the boundary by a system of receptors and ligands—EphB receptors and ephrinB ligands—that are both integral cell membrane proteins, and thus require direct cell contact for signaling. In this way, cell attachment-dependent contact induces signaling which leads to a temporary detachment, followed by reattachment and a next round of signaling. Such an attachment-detachment mechanism allows for cell migration along the boundary, while at the same time preventing invasion of the stationary tissue by the migrating one.

## Introduction

When Townes and Holtfreter [1] observed the sorting of mixed embryonic ectoderm, mesoderm, and endoderm cells, they proposed that this segregation of germ layers and the consequent self-assembly of the basic body structure was based on mutual “tissue affinities.” This concept was later refined into Steinberg's [2] Differential Adhesion Hypothesis, which posited that simple adhesion differences between cell populations are sufficient for their separation and their positioning relative to each other, explaining, for example, the arrangement of germ layers in amphibian embryos [3],[4]. However, if the boundary between two germ layers also serves for tissue translocation, more specialized tissue separation mechanisms may be required.

**Figure 1:**
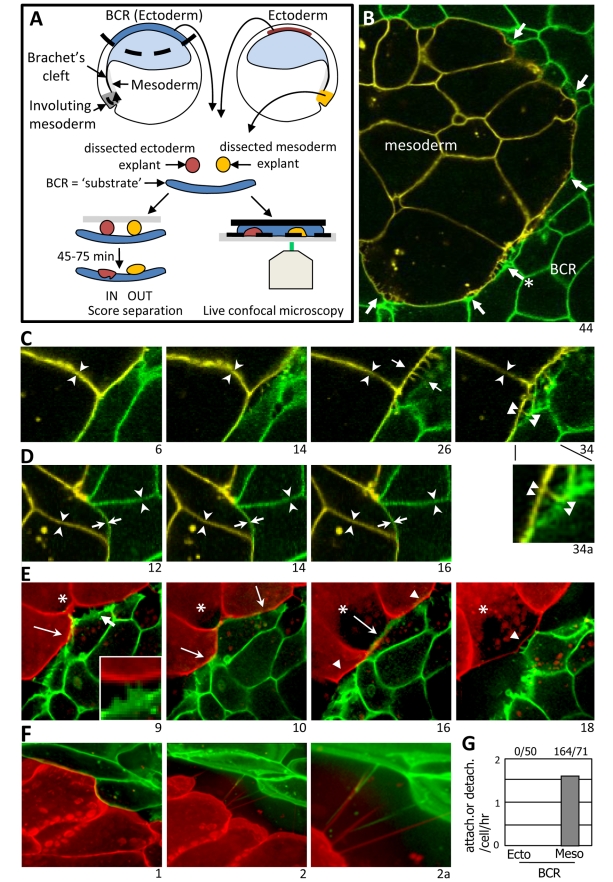
Cell repulsion at ectoderm-mesoderm boundary. (A) Tissue separation assay. Test explants are placed on BCRs, and the percentage remaining separate are scored after 45–60 min. Cell behavior at the boundary between tissues is examined by live confocal microscopy using test explants and BCRs with differently labeled membranes. (B–F) Live confocal microscopy. Test explants expressing mYFP (B–D) or mCherry (E, F) were combined with mGFP-expressing BCRs. Time between frames was 1 min for (B–D), 5 min for (E,F), frame number indicated. (B) Overview, mesoderm explant on BCR (Movie S1). Arrows show retractions. (C) Region indicated by arrow, asterisk in (B). Simultaneous retraction of mesoderm and BCR; a gap appears (14), spreads to upper right (26), then to opposite side (34). Protrusions extend into gap (26, short arrow). Insert 34a, double arrowheads: retraction fibers. Arrowheads: stable contacts within the tissues. (D) Ectoderm aggregate on BCR (Movie S2), showing stable contacts within the tissues (arrowheads) and at the interface between the two ectoderm explants (arrows). (E) Attachment/detachment of three mesoderm cells at BCR. Contact is first established by upper cell (long arrow, 9), then spreads through all cells (between long arrows, 10). Cells detach again from sides of frame (arrowheads, 16), central cell (*) (long arrow, 16) seems to resist and is last to detach (18). Arrow, insert in frame 9: cell protrusions. (F) Mesoderm on BCR. Initially in close contact (Frame 1), cells from both tissues retract (Frame 2) leaving retraction fibers behind (arrowheads, 2a). Four focal planes merged to capture entire fibers. (G) Quantification of attachment/detachment events. Numbers on top indicate total events/cells.

In the Xenopus gastrula, mesoderm translocates across the ectodermal blastocoel roof (BCR), and the boundary between these two germ layers, Brachet's cleft, must permit this movement yet prevent invasion of the BCR by the migratory mesoderm (Figure 1A). Interaction with a sparse network of fibronectin fibrils controls the motility of mesoderm cells, but their adhesion to the BCR is fibronectin-independent [5]. In fact, BCR and mesoderm cells are in direct contact [6], and the same adhesion molecules, C- and XB/U-cadherin, are expressed in both tissues (reviewed in [7]).

This ectoderm-mesoderm boundary has been established as a model for tissue separation. Properties of the mesoderm and the BCR that underlie their separation can be studied in an in vitro assay (Figure 1A) [5],[8]. The molecular control of separation behavior is partly known. Non-canonical Wnt signaling downstream of the Wnt receptor Xfz7 [9], interaction of Xfz7 with paraxial protocadherin (PAPC) and with the ankyrin repeat domain protein 5 (xANR5), and activation of RhoA and Rho kinase [10]–[12] are involved in the implementation of a proximal yet still unknown cellular mechanism that actually generates the boundary.

A candidate for this proximal mechanism is ephrin/Eph signaling. Eph receptor tyrosine kinases are subdivided into EphA and EphB subclasses and their membrane-linked ephrin ligands correspondingly into ephrinAs and ephrinBs. Within subclasses, binding appears promiscuous, although some Eph receptors have a higher affinity for specific ephrins. Receptor ligation and clustering initiates “forward signaling,” but receptor-ligand interaction can also stimulate “reverse signaling” downstream of the ephrin ligand. Ephrin/Eph signaling has been implicated in boundary formation under conditions where receptor and ligand are expressed in complementary patterns [13]–[18]. For example, a model was proposed for rhombomere-boundary formation based on repulsive Eph-ephrin signaling, acting in parallel with an Eph-dependent regulation of adhesion within the rhombomeres [19],[20]. However, direct observation of repulsive behavior at the boundary, similar to what is classically seen during neuron guidance, has not been attempted. In the early Xenopus embryo, expression of several Eph receptors and ephrins has been reported (reviewed in [21]). Their in vivo functions during gastrulation have not yet been established, although gain-of-function experiments indicate that they can regulate cell adhesion, migration, and sorting [22]–[24]. Thus, ephrin signaling is a prime candidate to mediate tissue separation in the early Xenopus embryo.

## Results

### Cells Display Sustained Attachment-Repulsion Cycles at the Ectoderm-Mesoderm Boundary

To examine cell contact dynamics at the boundary in living tissues, membrane-labeled mesoderm explants and ectodermal BCRs were combined, and the reconstituted boundary was observed by confocal microscopy (Figure 1 and Movies S1–S10). Contacts within each tissue appeared tight, as cells remained apposed and moved in concert (Figure 1B–D, arrowheads, Movies S1 and S2). The same behavior was seen at the boundary between BCR and ectoderm explants: cells re-established contacts within minutes and remained stably apposed throughout the experiments (up to 2 h) (Figure 1D, G, arrows, Movies S2 and S6). In contrast, contacts between mesoderm and BCR cells appeared dynamic (Figure 1C arrow, E, Movies S1 and S3) with frequent detachments followed by re-establishment of contacts (Figure 1G).

The frequency and length of detachment phases was variable. Also, separation was not uniform over the length of the boundary (see, e.g., Figure 1B,E, Movies S1 and S3). While in close contact, mesodermal cells appeared to adhere to the BCR. They moved in concert with BCR cells, and during subsequent detachment, taut retraction fibers often spanned the gaps between cells (Figure 1C, Frame Insert 34a; Figure 1F, Frame 2a; Movies S1 and S3). A detachment event typically spread along the boundary, where existing contacts seemed to resist retraction (see, e.g., Movie S1, and Figure 1C and E). Detached cells from either side of the boundary can emit protrusions which probe the cleft (Figure 1C, Frame 34; Figure 1E, Insert Frame 9; Movies S1 and S3). While repeatedly retracting and attaching, mesoderm cells can move along the BCR into gaps produced by other retracting mesoderm cells (Movie S3). This suggests a mechanism for the normal collective migration of mesoderm on the BCR substratum and is consistent with the co-existence of close adhesive contacts and gaps between mesoderm cells and the BCR at the ultrastructural level [6]. Altogether, it appears that contact-induced detachment between ectodermal and mesodermal cells is at the base of a tissue separation mechanism supportive of cell movement at the interface.

### In Vitro Activation of EphB Signaling Is Sufficient to Induce Tissue Separation

**Figure 2:**
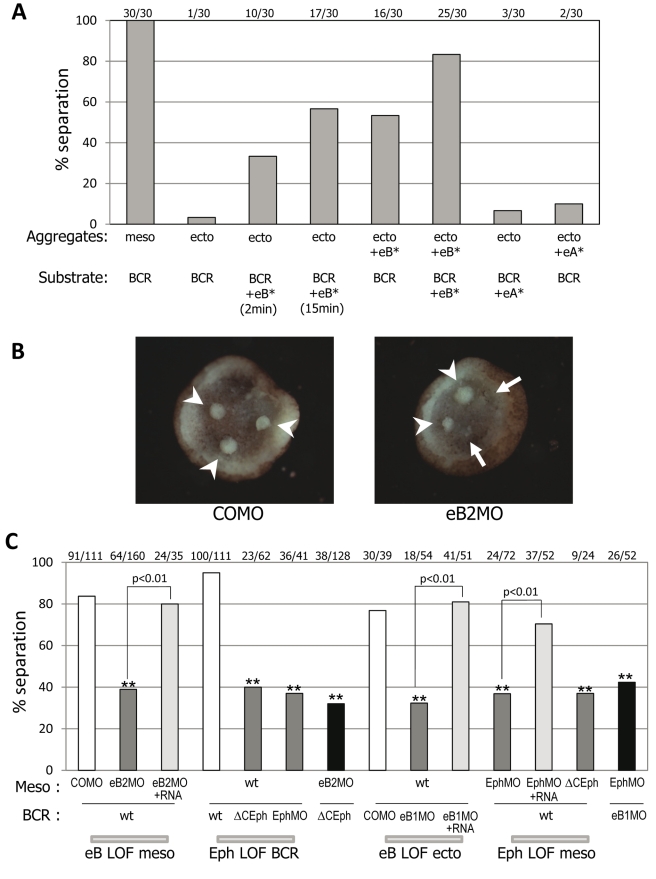
EphrinB/EphB signaling controls tissue separation. (A) Soluble ephrin B fragments induce tissue separation between ectodermal cells. Separation assay was performed after a short pre-treatment of the ectodermal aggregates (ecto) or of the BCR substrate with soluble, pre-clustered ephrinA- or ephrinB-Fc fragments (eA*/eB*). Unless stated otherwise, pre-treatment was for 15 min. Untreated mesoderm aggregates (meso) on untreated BCR were used as positive controls for maximal separation behavior. Untreated ectoderm explants rapidly sunk into untreated BCR. Strong separation was induced when either ectodermal aggregates or BCR were treated with eB*. Separation was particularly strong when both the test aggregates and the BCR were treated by performing the assay in eB* solution. eA* had no effect. (B,C) EphrinB/EphB signaling is required for tissue separation. (B) Separation assay: EphrinB depletion inhibits separation. Mesoderm test explants injected with control MO (COMO) remained on the surface of the BCR (arrowheads); of those injected with ephrinB2 MO (eB2 MO), 2 out of 4 invaded the BCR in the example shown (arrows). (C) Quantification of loss-of-function (LOF) experiments for ephrinBs and EphBs. Injections in mesoderm (Meso) or ectodermal BCR are indicated below bars for each tissue combination; wt, uninjected, ephrinB1,2 MO (eB1,2MO), EphB4 MO (EphMO), cytoplasmically deleted EphB (ΔCEph). Numbers of test explants remaining separated/total number of explants on top of bars. ** indicates *p*<0.01 compared to controls. Simultaneous depletion/inhibition in the mesoderm and the BCR (eB2MO/ΔCEph) did not increase the phenotype compared to eB2MO or ΔCEph alone (*p* = 0.2 and 0.3).

We tested the role of ephrins and Ephs on tissue separation in an in vitro assay (Figures 1A, 2B). Test explants were placed on a dissected BCR, and the percentage of explants that remain separate after 1 h was determined. In this assay, wild type ectoderm explants all sink into the BCR, while the majority of wild type mesoderm explants remain distinctly separated, i.e. on the surface of the BCR. Note that after 45–60 min, the reaction is complete: explants either have fully integrated (usually within 15 min for wild type ectoderm) or will remain definitively separated, implying that for individual explants the response is all or none. However, when the percentage of explants remaining separated in a given experiment is counted, the overall outcome is graded, which can be shown by increasing concentrations of interfering reagents (e.g., Figure S3) and/or increasing incubation times with activating Fc fragments (e.g., Figure 2A).

We first performed gain-of-function experiments on ectoderm tissues by treating them with activating, pre-clustered extracellular ephrin-Fc fusion polypeptides. While untreated ectodermal BCR explants readily reintegrated into BCRs, test explants incubated with ephrinB2-Fc tended to remain separate (Figure 2A). The same result was obtained by treating the substrate BCR instead of the test explants. Separation increased with incubation time, but could be detected as soon as 2 min after treatment (Figure 2A), indicating a rapid and direct cellular response. Activation was reversible, as a 30 min wash significantly decreased separation behavior (not shown). Separation was strongest when both the substrate BCR and BCR test explants were treated with ephrinB-Fc, over the whole duration of the assay, suggesting that the effect is not due to the establishment of a difference between two cell population but rather to a change in cell contact behavior. The response is specific for ephrinBs; ephrinA-Fc had very little effect (Figure 2A). These results imply that an EphB receptor is present on ectodermal cells and that its stimulation by ephrinB can immediately induce tissue separation. Consequently, we determined the expression pattern of all known ephrinB and EphB receptor isoforms in the gastrula by RT-PCR (Figure S1): all isoforms were expressed in all germ layers, although at different levels; as an exception, ephrinB3 was restricted to the ectoderm. Eph receptor-ephrin ligand binding is largely promiscuous, providing ample opportunity for redundancy. Moreover, Eph/ephrin signaling has previously been implicated in boundary formation in situations where receptor and ligand are expressed in complementary patterns in vivo (e.g., [25]–[27]) or under experimental conditions (e.g., [24],[28]). With co-expression of receptors and ligands in each of two adjacent tissues, the question arises whether Eph/ephrin signaling can nevertheless be employed for their separation.

### EphrinBs and EphB Function Are Required for Tissue Separation

To address this issue, we performed loss-of-function experiments using antisense morpholino oligonucleotides (MOs) against ephrinBs and EphBs to knock down putative crucial factors for boundary formation. Interfering with ephrin/Eph signaling led to severe developmental defects (Figure S2D–F). Most strikingly, analysis of early gastrulae revealed a strong reduction of Brachet's cleft (Figure S2A–C). While disruption of the cleft alone will affect gastrulation movements and produce shorter tadpoles (Figure S2E), ephrin/Eph signaling appears to disturb additional processes (EphB depleted embryos die before hatching, Figure S2F). By using the in vitro assay (Figure 1A) and by directly examining cell behavior, we isolated its specific function in tissue separation. Ephrin B1 and B2 MOs indeed inhibited tissue separation when injected either in the mesoderm or in the BCR (Figure 2B,C; Figure S3A,B; unpublished data), showing that both ephrins are required on both sides of the boundary. EphrinB1 MO was slightly more efficient in the BCR (unpublished data) and ephrinB2 MO in the mesoderm (Figure S3A,B), consistent with relative expression levels of the two ligands (Figure S1). Inhibition of separation shows dose-dependence before reaching a plateau at around 30%–40% (Figure S3B). Knockdown of ectoderm specific ephrinB3 caused a similar partial inhibition (Figure S4A). In all cases, separation was rescued by co-injection of corresponding wild type ephrinB mRNA (Figure 2C and Figure S4A). Inhibition was not significantly increased in double knockdown of ephrinB1 and B2 in the mesoderm (Figure S3A,B), and the same was true for triple knockdown of all ephrinB1–3 in the BCR (Figure S4A). We conclude that all ephrinBs expressed in a given tissue are required to a degree related to their relative expression levels, but simultaneous depletion is not sufficient for complete inhibition of separation.

We next tested whether ephrinB2, which is enriched in the mesoderm, could induce separation when overexpressed in the ectoderm. We found that a significant number (∼40%) of ectoderm explants now remained separated from wild type BCR (Figure S4B). However, overexpression of ephrinB1, which is already abundant in the ectoderm, had no effect (Figure S4B). Both constructs are strongly expressed (unpublished data) and effectively rescue normal separation behavior (Figure 2C). These results show that increasing ephrinB2 levels is sufficient to trigger separation and suggests that formation of the ectoderm-mesoderm boundary relies at least partly on the differences in ephrin composition observed between these two tissues (Figure S1).

Since ephrinB2 is required in the mesoderm, overexpression in the BCR of a cytoplasmically truncated form of the cognate receptor EphB4 (ΔC-EphB4) should compete for ephrin B2 binding with all endogenous Eph receptors and inhibit forward signaling in this tissue. Expression of ΔC-EphB4 in the BCR did indeed diminish separation, to a degree very similar to that obtained by ephrinB loss-of-function in the mesoderm (Figure 2C). EphB4 MO mimicked this effect, both in the BCR and in the mesoderm (Figure 2C), demonstrating that EphB4 is required. We conclude that the B family ligands and receptors are required in both ectoderm and mesoderm for tissue separation.

In the Xenopus gastrula, PDGF-A is expressed in the ectoderm and its receptor, PDGFR-α, in a complementary pattern the mesoderm. However, inhibition of PDGF signaling does not affect formation of the boundary (Damm and Winklbauer, submitted manuscript) or separation behavior in the BCR assay (Figure S4C). Likewise, interaction with the fibronectin-rich matrix on the BCR, which together with PDFG-A signaling determines the direction of mesoderm cell movement across the BCR [29], is not required for tissue separation [30], emphasizing the dominant role of EphB/ephrinB signaling in this process.

### Evidence for Two Anti-Parallel EphrinB/EphB Pathways Signaling Across the Ectoderm-Mesoderm Boundary

**Figure 3:**
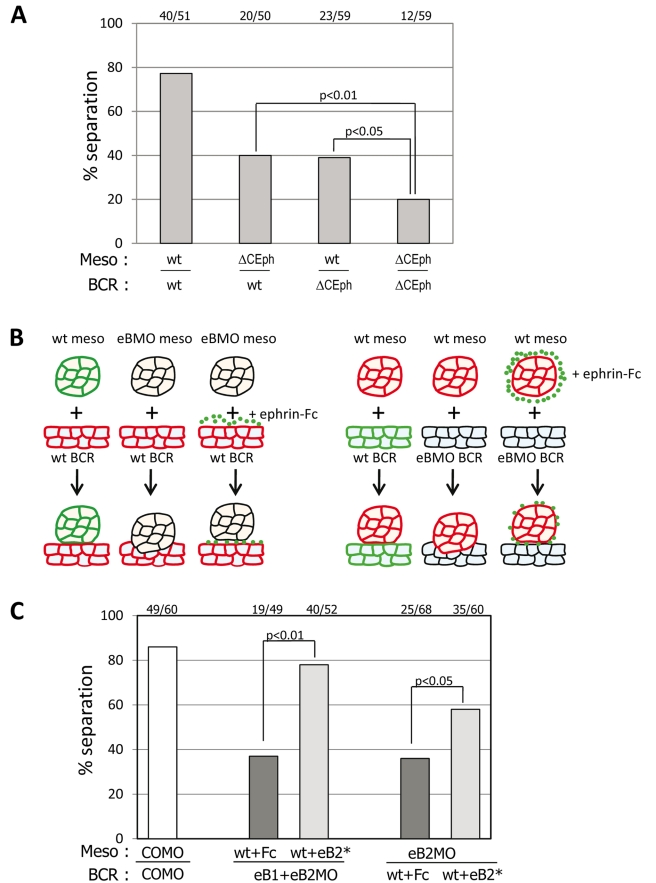
Forward signaling across the boundary is required in both apposed tissues. (A) Enhanced inhibition of separation by interference with EphB signaling in both tissues. ΔC-EphB was expressed in the mesoderm, in the BCR, or in both. Symbols as in Figure 2. (B–C) Rescue of separation by soluble ephrinB2-Fc fragment after ephrinB depletion. (B) Diagram of the experiment. Ephrins in one tissue and their cognate Eph receptors in the other tissue are represented as green and red plasma membranes, respectively. Ephrins were depleted in one of the tissues (eB1 and eB2 in the BCR and eB2 in the mesoderm, depletion symbolized by black membranes), and the signal was then restored at the surface of the other tissue by a 15 min treatment with ephrin-Fc fragment (eB2*, green dots) before assembly of the assay. (C) Quantitative data. Fc, control Fc fragments. Otherwise, symbols as in (B).

Our results suggest that ephrinB/EphB signaling occurs within each tissue on either side of the boundary, or that two anti-parallel pathways signal across the boundary. To distinguish between these alternatives, we performed a series of systematic ephrinB/EphB double knockdown/inhibition/rescue experiments. We first established that we maximally inhibited ligand or receptor activities: coinjection of ephrinB1 and B2 MOs indicated saturation of ephrinB inhibition (Figure S3A,B). Moreover, the degree of inhibition upon expression of ΔC-EphB could not be increased by increasing mRNA levels (Figure S3C). Thus, even when a pathway was maximally inhibited on one side, separation was only partially impaired. However, separation could be further reduced by interfering with ΔC-EphB4 on both sides of the boundary (Figure 3A and Figure S3C). Increased inhibition was also obtained by downregulating ephrinB and EphB activity simultaneously in the mesoderm (Figure S3B) or in the BCR (unpublished data), but not by downregulating ephrinBs in one tissue and EphB in the other (Figure 2C). Since these double inhibition experiments were preformed under conditions of maximal EphB interference (Figure S3C), ephrinB inhibition should not have had an effect if in the same EphB pathway. Thus, the simplest interpretation of our results was that separation is controlled by the additive activity of two antiparallel pathways signaling across the boundary. Each pathway involves ephrin ligands on one side and Eph receptors on the other side.

We tested this hypothesis more directly using ephrinB-Fc fragments (Figure 3B,C), which allowed us to stimulate these pathways specifically at the tissue surface, i.e. at the boundary. The rationale was to deplete ephrins in one of the interacting tissues, and then restore the forward signal by treating the other tissue with ephrin-Fc fragments to trigger activation of Eph receptors at its surface (Figure 3B). When we activated EphB signaling in mesoderm explants by treatment with ephrinB2-Fc (which binds to all EphB receptors) and placed them on ephrinB1/B2 depleted BCR, robust separation occurred (Figure 3C). Thus, direct activation of EphB receptors at the mesoderm surface can rescue separation from ephrin-depleted ectoderm, implicating forward signaling from the ectoderm to the mesoderm in tissue separation. The complementary experiment—ephrinB2 depletion in the mesoderm and EphB activation at the surface of the BCR—also resulted in a rescue of separation (Figure 3C), indicating that forward signaling from the mesoderm to the ectoderm is similarly active during tissue separation. The experiments show that separation can be restored by activating forward signaling directly at the surface of adjacent tissues. Thus, the separation phenotype induced by ephrinB loss-of-function can be fully accounted for by an inhibition of signaling across the boundary. Altogether, full tissue separation requires two forward signals, one triggered by mesodermal ephrins binding to the EphB receptors at the surface of the ectoderm and a second one depending on ectodermal ephrins interacting with Eph receptors of the mesoderm.

### EphrinB/EphB Signaling Is Required for Cell Detachment at the Boundary

**Figure 4:**
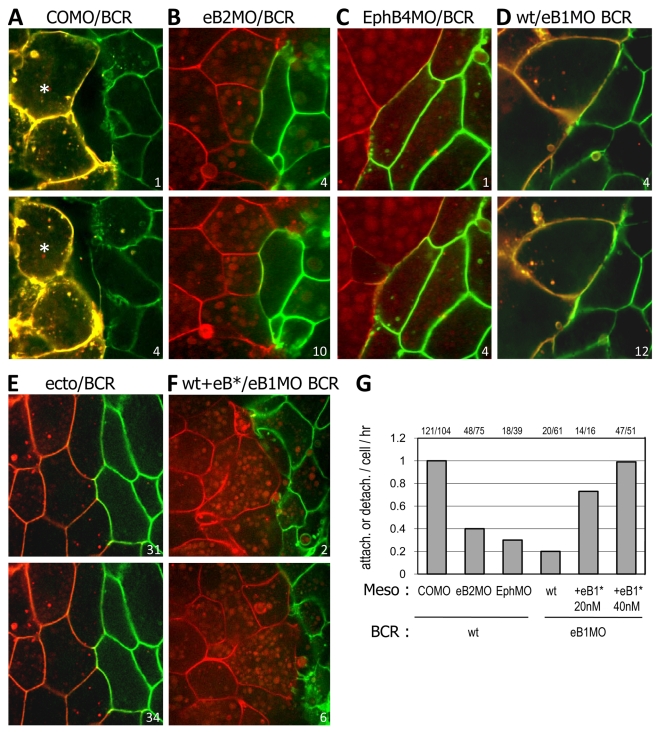
Ephrin/Eph loss-of-function inhibits repulsion at the boundary. (A–E) Effect of ephrin/Eph MO on repulsion. Time lapse spinning disc confocal microscopy using mesoderm explants expressing mCherry on mGFP-expressing BCRs. EphrinB2 MO or EphB4 MO in mesoderm (B,C), or ephrinB1 MO in BCR (D) inhibited repulsion compared to control MO (A). The interface between inhibited mesoderm explants and BCR resembled ectoderm-ectoderm contacts (E). (F) Incubation of mesoderm test explants with ephrinB1-Fc fragments rescues repulsion from ephrinB1-depleted BCR in a dose-dependent manner. (G) Quantification of attachment/detachment events per cell per hour. Numbers on top indicate total events/cells.

EphrinB or EphB knockdown impedes cell detachment at the boundary (Figure 4). Compared to controls (Figure 4A), ephrinB2 MOs in the mesoderm (Figure 4B) dramatically decreased the frequency of attachment/detachment events (Figure 4G). Often, mesoderm cells remained apposed to BCR cells for the whole duration of the recording (Figure 4B, Movie S4), similar to ectoderm aggregates (Figure 4E, Movie S6). Mesoderm and BCR cells moved in concert, indicating stable contacts (Movie S4). Detachment was similarly inhibited when ephrinB1 was depleted from the BCR, or EphB4 from the mesoderm (Figure 4C,D,G, Movie S5). Thus, signaling in both tissues is required for cell detachment at the interface. Attachment/detachment cycles were rescued after ephrinB1 depletion in the BCR by incubating wild type mesoderm test explants with increasing doses of soluble ephrinB1-Fc fragments (Figure 4F,G), demonstrating that this behavior is an immediate reaction to ephrin-Eph signaling at the boundary.

The experiments above show that direct ephrin activation at the tissue interface accounts for repulsion between ectoderm and mesoderm. In our time lapse recordings, repulsion is never observed within the tissues, but ephrin/Eph signals may nevertheless affect cell-cell adhesion [18],[19],[20],[22]. We evaluated the effect of Eph/ephrin loss- and gain-of-function on adhesion of ectoderm and mesoderm cells, using a classical reaggregation assay. Aggregation of dissociated cells started within minutes (unpublished data), and mesoderm had formed smaller aggregates than ectoderm after 1 h (Figure S5), consistent with mesoderm cells being less adhesive [31],[32]. EphrinB2 or EphB4 depletion had no effect on the rate of mesoderm cell aggregation (Figure S5), suggesting that ephrinB/EphB activity does not contribute to mesoderm tissue cohesion. In the ectoderm, however, corresponding ephrinB1/EphB4 depletion diminished aggregation. Overexpression of ephrinB2 had a similar, although more variable, effect (Figure S5). Thus, while ephrinB2 overexpression induces tissue separation and ephrin/Eph depletion inhibits separation, both treatments reduce cohesion within the ectodermal tissue. Altogether, putative effects of ephrins and Eph receptors on cell-cell adhesion within tissues are not correlated with their roles in cell detachment at the tissue boundary.

### RhoA and Rac GTPase Mediate Tissue Separation Downstream of Ephrin/Eph in Both Mesoderm and BCR

**Figure 5:**
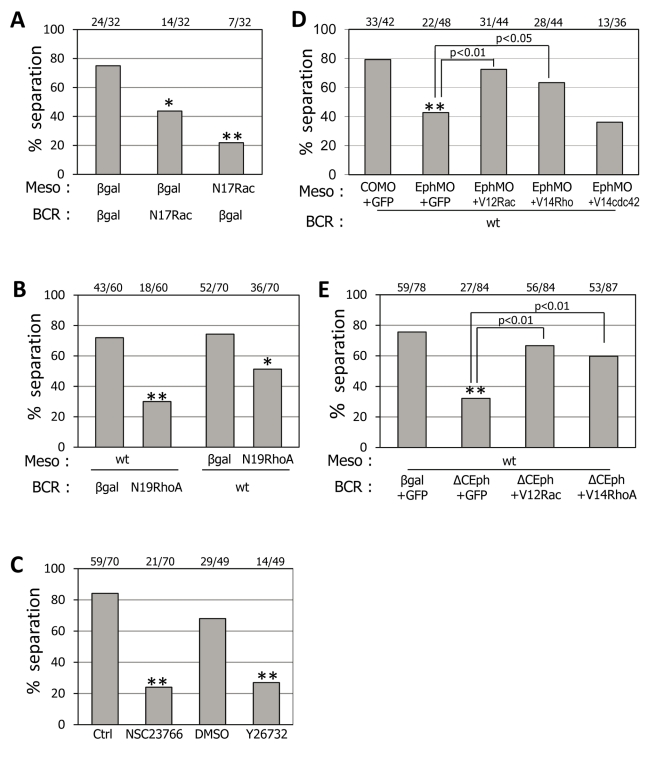
Involvement of RhoA and Rac downstream of ephrin/Eph signaling. (A–C) Rac and RhoA activities are required for tissue separation. (A, B) Expression of dominant negative forms N17Rac and N19RhoA either in the mesoderm test aggregates or in the BCR blocked separation. (C) Separation was strongly inhibited when assays with wt mesoderm and wt BCR were performed in the presence of the Rac inhibitor NSC23766 (18 µM) or the Rok inhibitor Y26732 (50 µM). (D–E) Rac or RhoA activation can rescue loss of ephrin signaling. Signaling was inhibited by injecting EphB4 MO (D) in the mesoderm or expressing ΔCEphB in the BCR (E). In both cases, separation was rescued by co-expression of constitutively active V12Rac or V14RhoA. Constitutively active cdc42 (V14cdc42) had no effect (D).

RhoA and Rac are well-established downstream effectors of Ephrin/Eph signaling that modulate cytoskeletal dynamics. RhoA activity in the mesoderm had been implicated in separation behavior [11], but the role of RhoA in the BCR and of Rac in both tissues has not yet been addressed. We systematically tested the effects of manipulating RhoA or Rac function. Dominant negative N19RhoA and N17Rac both inhibited separation when expressed in either of the two tissues (Figure 5A,B). Because expression of constructs interfering with RhoA and Rac function may also have long-term indirect effects, we complemented these data with experiments using specific soluble inhibitors of Rac and of Rho-associated kinase, a direct target of RhoA (Figure 5C). A short incubation with these inhibitors was sufficient to cause mesoderm test explants to integrate into the BCR, thus mimicking the effect of the dominant negative GTPases. This immediate response to the inhibitors suggests that RhoA and Rac activities are directly required during the separation process.

We next asked if RhoA/Rac activation could rescue separation when ephrin/Eph signaling is impaired. Since we had demonstrated forward signaling in both directions, we inhibited signaling on the Eph receptor side, by injection of EphB4 MO in the mesoderm and ΔC-EphB4 in the BCR, and tested the effect of constitutively active forms of RhoA and Rac (V14RhoA,V12Rac), expressed at low levels in the same tissue. In both cases, separation could be efficiently rescued by both V14RhoA and V12Rac (Figure 5D,E). A weak rescue was also observed upon overexpression of wild type RhoA and Rac (Figure S6B). Constitutively active Cdc42 was unable to rescue separation (Figure 5D). We conclude that RhoA and Rac, but not Cdc42, function downstream of Eph signaling on both sides of Brachet's cleft. Since RhoA has also been proposed to act downstream of Xfz7/PAPC/xANR5 in the mesoderm to regulate tissue separation [11], we asked whether co-expression of Xfz7 and PAPC could rescue separation in ephrinB2+EphB4 MO-injected mesoderm. We did not observe any rescue (Figure S4C), indicating that ephrinB/EphB signaling acts downstream of or in parallel to Xfz7/PAPC.

**Figure 6:**
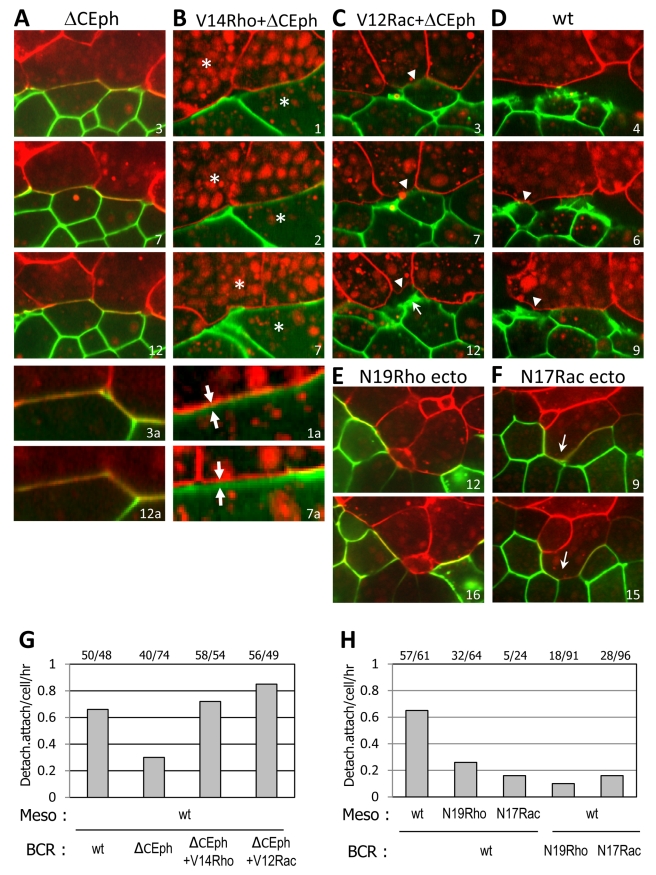
Effect of Rho/Rac modulation on cell behavior at the boundary. Live confocal microscopy of mesoderm explants expressing mCherry combined with mGFP-expressing BCRs. (A–F) Selected frames from Movies S7 and S8. (A–D) Inhibition of repulsion by ΔCEph in the BCR (A) is recued by co-expression of constitutively active forms of RhoA (V14Rho) (B) and Rac (V12Rac) (C). (D) Control wild type mesoderm and BCR. Arrowheads in (C) and (D) point at mesoderm cell membranes that are initially in contact with BCR cells and detach in subsequent frames. The arrow in Frame 12 of (C) points to a protrusion emanating from the BCR cell after detachment. In the example shown in (B) cells remain detached for a prolonged period (compare the two parallel but separated membranes in the enlarged field B1a and B7a with the merged signals from the closely apposed membranes for ΔCEph alone, A3a, and A12a). Asterisks indicate the relative positions of two mesoderm and ectoderm cells sliding along the boundary, and the antiparallel arrows show the relative translation of these cells. This phenotype is observed in both V14RhoA and V12Rac rescues. (E–F) Inhibition of repulsion by dominant negative forms of RhoA and Rac. N19RhoA and N17Rac caused ectoderm and mesoderm cells to remain stably attached. Note disruption of the boundary through intercalation of cells from both tissues (F, arrows). (G, H) Quantification of attachment/detachment events per cell per hour. Numbers on top indicate total events/cells.

We next examined the effect of RhoGTPases activity on repulsion at the cleft by live confocal microscopy (Figure 6). When dominant negative forms of RhoA or Rac were expressed in the BCR or in the mesoderm, the frequency of attachment/detachment was strongly reduced in all cases (Figure 6H). Most cells established stable contacts between ectoderm and mesoderm that were indistinguishable from contacts within tissues (Figure 6E,F). Conversely, when activated forms of RhoA or Rac were co-expressed with dominant negative EphB receptor, the rescue of tissue separation (Figure 5D,E) was paralleled by a rescue of detachment at the boundary (Figure 6A–D,G, Movies S7 and S8). However, compared to controls, repulsion at the boundary appeared more transient. Although the membranes of abutting cells often remained close to each other for prolonged periods, they were clearly detached, and cells were able to slide along the boundary (Figure 6B), indicating that they had failed to re-establish contacts. It appeared as if cells were locked in a detached state by active RhoA or Rac, but could not fully retract under these conditions.

Our results imply that RhoA and Rac should be locally activated at ectoderm-mesoderm contacts in an Eph signaling-dependent manner. We investigated the subcellular localization of active, GTP-bound GTPases in the mesoderm by expressing the GTPase-binding domains (GBD) of N-Wasp (a target of Cdc42/Rac) or Rhothekin (a target of RhoA) fused to GFP. These mesodermal test explants were combined with BCRs expressing mCherry and examined by spinning disk confocal microscopy. While these constructs can act as inhibitors of their respective GTPases, at low expression levels they are expected to accumulate at sites of high concentrations of active GTPases [33]–[35].

**Figure 7:**
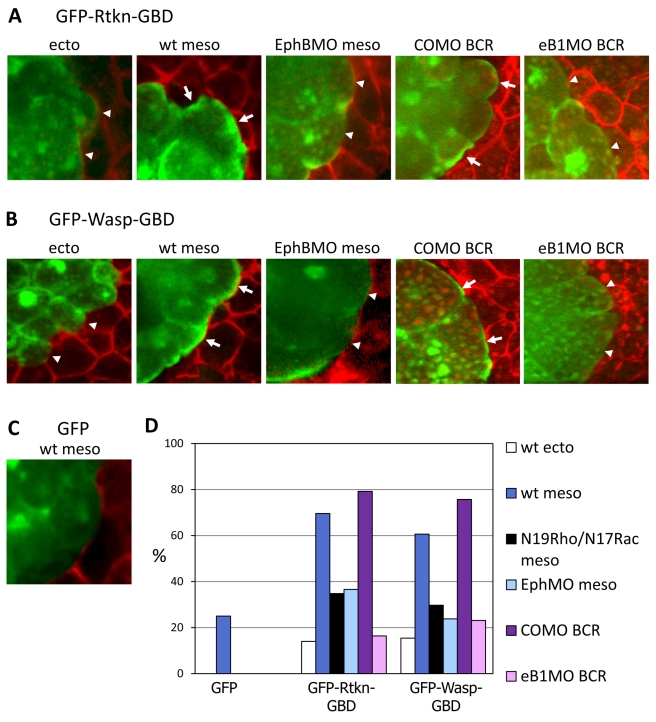
Subcellular localization of activated GTPases at the boundary. Explants expressing GFP-Rhothekin-GBD (A), GFP-Wasp-GBD (B), or control GFP (C) were combined with mCherry expressing BCRs and analyzed by live confocal microscopy. Both GDBs accumulated preferentially at sites of contact with the BCR in wt mesoderm (arrows), but not in wt ectoderm (arrowheads). Control GFP in wt mesoderm did not accumulate at the boundary. EphB4 depletion (EphMO) in the mesoderm or ephrinB1 depletion (eB1 MO) in the BCR both strongly decreased accumulation at most contact sites (arrowheads), similar to expression of dominant negative forms of RhoA (N19Rho) for GFP-Rhothekin-GBD or Rac (N17Rac) for GFP-Wasp-GBD. (D) Quantification: percentage of cells showing accumulation at contact sites with the BCR.

We observed accumulation of both GBDs at the ectoderm-mesoderm boundary (Figure 7A,B, arrows). Signal intensity was generally higher there than at cell contacts within the explants. We quantified the GBD distribution in all cells which were in contact with BCR cells (Figure 7D). While in controls, BCR-BCR contacts showed accumulation in less than 20% of the cells, about 60%–80% of mesoderm cells contacting the BCR scored positive for a high fluorescent signal, for both Wasp and Rhothekin GDBs. This was a significant increase (*p*<0.001) compared to GFP alone. EphB4 MO seemed to inhibit GBD accumulation at boundary contacts (arrowheads): the frequency of accumulation decreased to 30%–40% (*p*<0.001). A similar frequency was obtained for mesoderm cells expressing dominant negative GTPases (Figure 7D). We also observed a dramatic decrease in Wasp/Rhothekin-GBD boundary localization when ephrin B1 was depleted in the adjacent BCR (frequency around 20%, *p*<0.0001 compared to control MO) (Figure 7A,B,D). We have not yet been successful in performing the reciprocal experiment, i.e. imaging GBDs in the BCR. This tissue appears to tolerate expression of GBDs less. However, the data from mesoderm explants demonstrate an Eph signaling-dependent activation of GTPases at the boundary.

**Figure 8:**
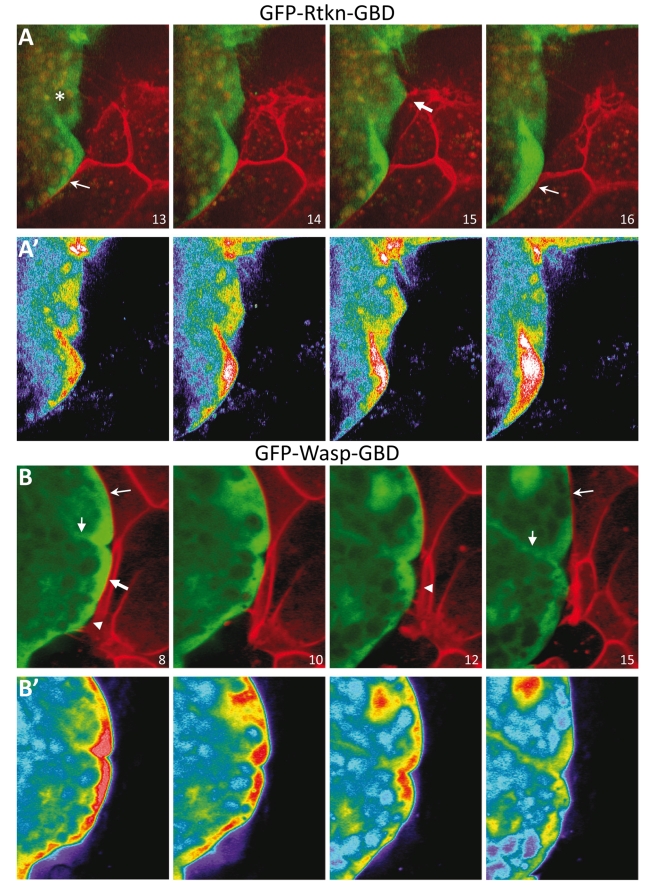
Dynamic activation of RhoGTPases at the ectoderm-mesoderm boundary. Explants expressing GFP-Rhothekin-GBD (A) and GFP-Wasp-GBD (B) were combined with mCherry expressing BCRs. Selected frames from Movies S9 and S10. Time between frames was 2 min for (A) and 1 min for (B). (A',B') Pseudocolors for the GFP signal intensity. (A, A') The mesoderm cell labeled with an asterisk establishes contact with a BCR cell in Frames 14 and 15 (arrow), then retracts in Frame 16. GFP-Rhothekin-GBD concentrates near the site of contact in Frames 14 and 15, and the signal decreases after detachment (Frame 16). A second cell in the lower part of the field accumulates progressively higher levels of GFP-Rhothekin-GBD but fails to retract. (B,B') Two cells are initially in close contact with the BCR. While the upper cell maintains the contact throughout this sequence (Frame 13, thin arrow), the lower cell (thick arrow) detaches progressively starting from the lower edge (arrowheads). The intense GFP-Wasp-GBD signal at the sites of contact (Frame 8) dissipates in both cells (Frames 10–15), while the signal in the cytoplasm and near the membrane separating both cells (small arrows) remains relatively constant (small arrow Frames 8 and 15).

Time lapse microscopy of mesoderm cells expressing Wasp/Rhothekin-GBDs revealed that accumulation at the boundary was highly dynamic. As expected from the large variation in contact time and area (see above), and from the superposition of detachment and protrusion formation, we observed fluctuations that spanned a large range of intensities and were highly variable in frequency (Movies S9 and S10). Drastic changes often occurred between two frames, i.e. in less than 2 min, in agreement with a fast dynamics of GTPase signaling. Despite this variation, a correlation could nevertheless be seen between retraction and signal decay. We analyzed cells for which we could unambiguously determine a transition from intimate contact with the BCR to detachment. In almost all cases (14/16 cells for WaspGBD and 13/14 cells for RtknGBD, from four independent experiments) the membranes of mesoderm cells contacting the BCR showed significant accumulation of the GFP construct, which decreased once the cell had detached (Figure 8). Generally, retractions and GFP decay both happened from one frame to the next (e.g., Figure 8A). In some cases, retractions spanned several frames, which then correlated with a slower decay of the GFP signal (e.g., Figure 8B). Some of the changes in signal intensity that were observed in non-retracting cells may be due to small detachments not detectable at the resolution of the GFP-GBD signal. Also, GTPase activation events may simply not be successful in triggering detachment. Note that the fluctuations occurring asynchronously in neighboring areas (Figure 8A, small arrows). The signal from mesoderm-mesoderm (Figure 8B, thin arrows) contacts served an internal control for the absence of photobleaching in these recordings.

As for putative downstream targets of the GTPases, we visualized cytoskeletal F-actin, phospho-nonmuscle-myosinII, and microtubules. We did not detect any significant accumulations or depletions at the boundary for microtubules and P-myosin (unpublished data). F-actin was enriched at the boundary, and this was dependent on ephrin-signaling (Figure S7). However, the pattern did not fully correlate with separation behavior, as a significant decrease was observed in the presence of N17Rac but not of N19Rho. These data indicate that the cytoskeleton is indeed modulated at the boundary, although tissue separation must be mediated by processes that cannot be distinguished at the level of global F-actin distribution. They also suggest that Rac and RhoA have distinct effects downstream of ephrin signaling, although we did not detect evidence for synergy between RhoA and Rac at the level of separation behavior (Figure S6).

## Discussion

**Figure 9:**
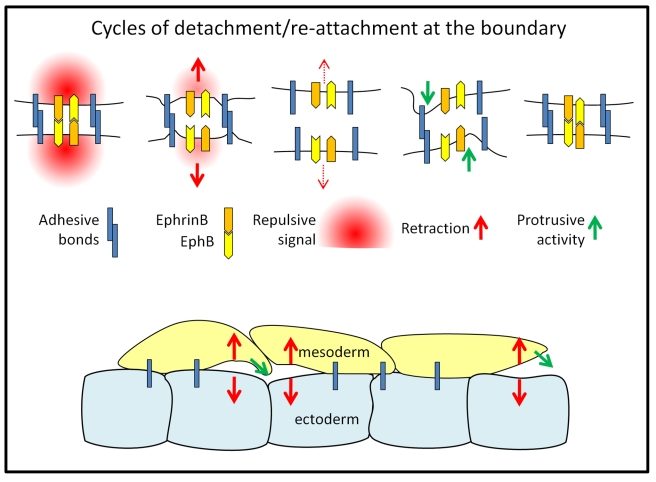
Model for tissue separation. Cells at boundary alternate between attachment and detachment: contact at boundary triggers signaling through membrane-bound ephrinB and EphB receptors, which induces repulsion. Once cells are apart, the signal decays, cells emit protrusions, and re-establish contacts. This prevents mesoderm cells from invading the BCR but allows them to use the BCR as substrate for migration.

Previous attempts to explain the separation of ectoderm and mesoderm [3],[4],[36] have been based on a thermodynamic model involving the minimization of tissue surface free energy. It assumes that two respective cell types are intrinsically different in terms of cell adhesiveness or cortical tension, and that this difference can drive cell sorting, boundary formation, and tissue positioning, analogous to the phase separation of immiscible fluids [2],[36]–[38]. Here we have shown evidence for a different model, in which signaling across the ectoderm-mesoderm boundary is crucial to locally regulate cell detachment and eventually tissue separation. Thus, although all cells of the two populations have the potential to form a cleft-like boundary if juxtaposed, acute separation behavior is not based on permanent adhesion differences between cells, but on transient contact-dependent reactions. The resulting, alternating phases of adhesion and repulsion appear to be part of a self-regulating loop (Figure 9). The spreading of an adhesion zone brings ephrins and Ephs in contact, inducing a repulsion signal. Once cells are apart, the signal decays, cells start to explore the intercellular space created at the boundary, and eventually re-establish adhesion. This mechanism prevents mesoderm cells from intruding into the BCR while providing necessary substratum contacts for migration.

Cell detachment is restricted to the tissue interface, and although various ephrins and their cognate receptors are coexpressed within each tissue, they do not lead to overall mutual cell repulsion and hence tissue dissociation. One possible explanation is suggested by our reaggregation experiments which show that EphB and ephrinB are required for normal ectoderm cohesion. This result is consistent with the fact that Eph/ephrin interaction between cells can mediate not only repulsion but also adhesion. The switch between these opposite responses depends on ephrin/Eph density, with adhesion being generally favored at low levels [39],[40], reviewed by Poliakov et al. [41]. In addition to expression levels, the different affinities for each other of the various Eph and ephrin subtypes [16] are likely to influence the strength of the response. Coactivation of EphB and ephrinB within the same cell can also induce an adhesive instead of a repulsive response [42], a mechanism that may likewise vary for different subtypes. In fact, our observation that overexpression of the mesodermally enriched ephrinB2 in the ectoderm is sufficient to trigger separation, but that ephrinB1 is unable to do so, suggests a surprising degree of specificity. This leads us to propose that the subtypes and levels of ephrins and Eph receptors expressed in each of the two tissues may be the main determinant for tissue separation. Since the ectoderm and the mesoderm express similar yet distinctly different mixtures of receptors and ligands, it is conceivable that the sum of interactions within each tissue is adhesive, while interactions across the boundary result in repulsion. However, in contrast to the ectoderm, no clear effect of Eph/ephrin function on tissue cohesion was observed for the mesoderm in our assay; possibly, the tissue is close to the transition point between adhesion and repulsion. Parallel mechanisms could further attenuate EphB/ephrinB signals within the mesoderm or enhance repulsion at the boundary. A candidate would be Xfz7/PAPC signaling, which is sufficient to induce separation in the ectoderm [11], and functions upstream or in parallel to EphB.

Importantly, regulation of tissue cohesion by EphB/ephrinB function seems to be independent of the control of tissue separation. In the ectoderm, ephrinB2 overexpression which triggers separation and ephrin/Eph depletion which inhibits it both decreased reaggregation, and in the mesoderm, inhibition of separation behavior is not associated with a change in overall cell-cell adhesion. This is consistent with other results indicating that changes in global adhesive strength do not necessarily affect tissue separation [43],[44]. The results indicate also that ectoderm-mesoderm separation is sensitive to the detailed expression pattern of Eph receptors and ligands, which is more complex than the classical complementary pattern, but may determine boundary formation in an essentially similar manner.

Ephrin/Eph-mediated boundary formation often involves the complementary expression of receptors and ligands in adjacent tissues and bidirectional signaling (e.g., [28]). This leads to rapid, large-scale changes in downstream pathways which differ in forward- and reverse-signaling cells [45]. Nevertheless, unidirectional signaling can be sufficient for cell segregation. Thus, in the zebrafish embryo, formation of the gap between adjacent somites depends on Eph forward signaling, while the ephrin reverse signal is dispensable [46]. Surprisingly, the same process using the same receptor and ligand isoforms requires ephrin reverse signaling, but not an Eph forward signal, in the chick embryo [47]. We found that similar to zebrafish somite segmentation, forward signaling is essential for ectoderm-mesoderm separation, and that two antiparallel forward signals are sufficient for complete repulsion at the boundary.

The demonstrated requirement for both RhoA and Rac in tissue separation is consistent with their known functions in Eph/ephrin mediated repulsion. Thus, the termination of EphB-ephrinB interaction, required for repulsion, involves endocytosis of receptor and ligand [48], which in turn depends on Rac function downstream of EphB [49]. RhoA and Rho kinase activation downstream of an EphB receptor can be mediated by the adaptor protein, Dishevelled, and underlies experimentally induced cell sorting of Xenopus gastrula ectoderm cells [24]. The local activation of Rac and RhoA at the ectoderm-mesoderm interface agrees with the notion that Eph/ephrin signaling is activated strongly at the boundary only, despite the widespread co-expression of receptors and ligands.

It is worth noting that despite the very low levels used in our experiments, each of the constitutively active RhoA and Rac construct could efficiently rescue separation. While this may be due in part to the potency of these forms, the cellular phenotypes produced by their overexpression appeared relatively mild. This was illustrated by the fact that even though the boundaries often appeared locked in a separated state upon rescue, attachment/detachment cycles were still observed occasionally. We hypothesize that in our inhibition experiments, ephrin/Eph signaling is decreased below the threshold required to maintain separation, but not completely abolished. Low amounts of active GTPases are then sufficient to boost the process and restore repulsion, while still allowing subsequent re-attachment. Alternatively, low levels of residual ephrin/Eph signaling may be sufficient to deliver exogenous active RhoA and Rac to their respective targets, though not to activate the endogenous GTPases sufficiently. The weak rescuing effects of wild type Rac and RhoA are consistent with both possibilities. In any case, the process of tissue separation appears particularly sensitive to small positive or negative changes in RhoGTPase activity, while larger alterations are required to affect other processes known to depend on such activities, in particular cell-cell adhesion.

Cleft-like boundaries based on attachment/repulsion cycles could generally be a requirement for the migration of cells across the surface of an adjacent tissue. A cleft-like ectoderm-mesoderm boundary is also seen in the zebrafish gastrula [50], and migration of mesoderm cells across the ectodermal layer occurs in Drosophila gastrulation [51]. It would be interesting to see whether the ephrinB/EphB mechanism of tissue separation is conserved in these cases. In contrast to the sparse, cell-permeable network of fibronectin fibrils that covers the ectodermal BCR of Xenopus, mouse or chicken gastrulae possess a well-developed basal lamina which physically separates ectoderm from mesoderm, potentially rendering germ layer separation independent of a cell repulsion mechanism [52]. Indeed, mouse ephrinB1, ephrinB2, and EphB4 null mutants apparently gastrulate normally [53]–[56]. After gastrulation, numerous mass cell migration events take place, for example neural crest migration, which in principle could also employ the mechanism presented here. Finally, deposition of an extracellular matrix seems to require free tissue surface[57],[58], and initially separating rhombomeres or somites by a repulsion cleft could be the first step in the boundary maturation process which ends with a matrix-filled space between tissue blocks.

## Methods

### Plasmids and Morpholino Antisense Oligonucleotides

Plasmids and morpholino oligonucleotides (Genetools) are listed in Supporting Information section.

### Recombinant Proteins

Recombinant mouse EphrinB2/FC chimera (R & D systems) comprising the extracellular domain of mouse ephrinB2 fused to C-terminal 6X histidin tagged Fc region of human IgG were pre-clustered with anti-human Fc antibody (Jackson ImmunoResearch Laboratories) at a 1∶2 ratio in MBSH [5] and incubated 1 h before application.

### Injections

mRNAs were synthesized according to manufacturer instructions (mMessage mMachine kit, Ambion). MOs and mRNAs were injected animally in the two blastomeres of 2-cell stage embryos for ectoderm/BCR tissues, and equatorially in the two dorsal blastomeres of 4-cell stage embryos for mesoderm explants, at amounts listed in Text S1.

### In Vitro Separation Assay

The assay was performed as described [21]. Mesoderm explants were dissected from the lower lip region before the start of involution, as described [9]. For in vitro activation of Eph receptors, explants or BCRs were pre-incubated with preclustered ephrinB2-Fc fragments (40 nM in MBSH) for 15 min at room temperature. For the statistical analysis, results were compared using the paired sample Student's *t* test, taking each assay as an experimental unit. Thus each experiment was scored based on the percentage of test explants remaining separate (i.e., it is a graded response).

### Confocal Microscopy

Explants from embryos injected respectively with 2×200–400 pg mGFP, mYFP, or mCherry mRNA were mounted in a dish with a bottom cover slip. For Figure 1A–C and Movies S1 and S2, time lapse recordings were acquired using a Zeiss LSM510 with a 40× Neofluar NA = 1.3 oil objective. GFP and YFP were excited with the 477 and 514 argon laser lines. Dicroïc and emission filters were HFT477, BP500/20 for GFP, and HFT514, LP530 for YFP. In other experiments, a Quorum technologies WaveFX spinning disc confocal mounted on an automated DMI6000B Leica microscope was used, with a 40× HCX PL APO CS, NA = 1.25 oil objective. GFP and Cherry were excited with 491 and 561 nm diode lasers. Images were collected with EM CCD 512X512 BT camera and controlled with Improvision Volocity 3DM software. Image processing was performed with Metamorph (Universal Imaging Corporation) and Adobe Photoshop7 software. For phalloidin staining, explants were fixed in 4% formaldehyde in MBSX for 10 min, followed by permeabilization (1% formaldehyde, 0.1% TritonX100), 1 h incubation with blocking buffer (10% sheep serum), and overnight incubation with 2 U/ml Alexa488-phalloidin (Invitrogen) in 10% sheep serum. After three washes in PBS and addition of antifade reagent (Slowfade Gold, Invitrogen), samples were examined using the spinning disc microscope.

### Re-aggregation Assay

Mesoderm and inner layer ectoderm were dissociated in alkaline calcium-free buffer (88 mM NaCl, 1 mM KCl, 10 mM NaHCO_3_, pH 9.3). Dissociated cells were transferred to agarose-coated Petri-dishes in normal MBSH and incubated for 1 h under mild rotation (10 rpm) on an orbital shaker. Images of the whole area where aggregation occurred, i.e. including all single cells and all aggregates, were taken under a dissecting microscope at a 12.5× magnification using a Micropublished RTV3.3 camera (Qimaging) and were analyzed for object size using ImageJ software. Two parameters were measured, average object area and area/perimeter ratio. Results of five independent experiments were normalized using wild type ectoderm as reference (1.0).

## Supporting Information

Figure S1 Expression ephrinBs and EphBs in the three germ layers at early gastrula stage. RT-PCR was performed using mRNA extracted from ectoderm, dorsal mesoderm, and endoderm tissues dissected at stage 10.5. Loading was equalized by comparing levels of FGFR in the three tissues (unpublished data). Two independent experiments showed identical patterns of expression.
(2.25 MB TIF)

Figure S2 Whole embryo phenotypes for ephrinB2 and EphB4 depletion. EphrinB2 (eB2) and EphB4 MOs were injected in the two blastomeres of the two-cell stage embryo. Embryos were fixed at the early gastrula stage and bisected sagitally (A–C) or allowed to develop until early tadpole stages (D–F). (A–C) Arrows point to both ends of Brachet's cleft.
(3.38 MB TIF)

Figure S3 Multiple interference with ephrinB1, B2, and EphB. (A) Single and double EphrinB1 and B2 knockdown in the mesoderm by injection of various amounts of eB1 and eB2 MOs. Single MOs caused significant separation, demonstrating that both ephrins are required. Inhibition was dose dependent. It was not increased by co-injection of eB1 MO and eB2 MO, even with the highest amounts of MO, indicating that mesodermal ephrins contribute only partly to separation. (B) Simultaneous injection of EphB4 MO with eB2 MO or eB1+eB2 MO caused stronger inhibition of separation compared to single EphB4, eB2, or eB1+eb2 MO injections. (C) EphB inhibition by expression of dominant negative ΔC-EphB. EphB activity is required in both ectoderm and mesoderm. Inhibition by ΔC-EphB was dose dependent but reached a maximum at 400 pg. A significantly stronger effect was obtained by simultaneous interference in both tissues, both with levels yielding maximal (400 pg) or submaximal inhibition (150 pg).
(0.80 MB TIF)

Figure S4 (A) Interference with ephrin B3 in the BCR. Injection of eB3 MO (40 ng) in the BCR caused inhibition of separation (*p* = 7.40E-07). The degree of inhibition upon triple injection of eB1, eB2, and eB3 MO was similar to eB1 MO alone (see Figure 2C). Separation was rescued by co-injection of eB3 mRNA. (B) EphrinB2 overexpression is sufficient to induce separation behavior in the ectoderm. Embryos were injected with ephrinB1 or ephrinB2 mRNA (500 pg/injection). EphrinB2 induced separation (*p* = 0.01) while ephrinB1 had no effect (*p* = 0.24). (C) Effect of PDGF and Fz/PAPC signaling. Treatment of mesoderm explants with PDGF receptor kinase inhibitor AG1296 (10 µM) does not inhibit separation. Expression of Fz7 and PAPC does not rescue separation when co-injected with eB1 and EphB4 MOs.
(0.43 MB TIF)

Figure S5 Reaggregation. Dissociated ectoderm and mesoderm cells were left to reaggregate for 1 h. (A–D) Reaggregated wild type ectoderm (A), wild type mesoderm (B), ephrinB1-depleted ectoderm (C), and ephrinB2 overexpressing ectoderm (D). (E) The degree of reaggregation was determined using two criteria: the average particle size, reflecting the extent of aggregation, and the total area/perimeter ratio, which integrates both the size of the aggregates and their degree of compaction (single cells and small aggregates have a large area/perimeter ratio, large round aggregates have a minimal perimeter, thus a higher area/perimeter ratio). Results from individual experiments were normalized using wild type ectoderm as reference (1.0) to account for batch-to-batch variation. Both parameters gave similar results, and the same trend for each of the condition was observed at earlier time points (unpublished data): mesoderm reaggregated less rapidly than ectoderm. EphrinB1 and EphB4 depletions decreased ectoderm reaggregation. EphrinB2 overexpression lead to a similar, although more variable, inhibition. EphrinB2 and EphB4 depletion had no effect in the mesoderm.
(1.90 MB TIF)

Figure S6 Combined RhoA/Rac interference and rescue with wild type RhoA and Rac. (A) Dominant negative N19RhoA and N17Rac (100 pg mRNA) were expressed alone or in combination in the BCR. Double inhibition of RhoA and Rac did not significantly enhance inhibition of separation (data from six independent experiments). (B) ΔCEph was expressed alone or with wild type RhoA or Rac. Inhibition of separation by ΔCEph is weakly rescued by wild type RhoA and Rac. Rescue was not enhanced by simultaneous expression of both RhoA and Rac. Data were pooled from three experiments with doses of 100 pg and three experiments with doses of 200 pg RhoA or Rac mRNA. The strength of rescue was similar at both doses.
(0.86 MB TIF)

Figure S7 Eph-dependent F-actin accumulation at the mesoderm/BCR boundary. Mesoderm explants were combined with BCRs manipulated by injection of ΔCEph, N17Rac, N19RhoA mRNAs, or eB1 MO. Sample were fixed and stained with Alexa488-phalloidin and analyzed by confocal microscopy. Stacks of five focal planes (2 µm) were merged, and relative intensity levels were compared using pseudocolors. (A) Boundary between mesoderm and BCR. F-actin is concentrated at cell cortex, with BCR cells having a stronger signal than mesoderm cells. The boundary (arrows) showed F-actin accumulations (thick arrows) similar to those found at some tri-cellular junctions in the BCR (arrowheads). (B) Mesoderm combined with ΔCEph-expressing ectoderm. F-actin accumulation at the mesoderm/BCR interface (arrows) was weaker than in controls. (C) Quantitation of F-actin accumulation at the boundary. Using pseudocolors, BCR cells were scored for stronger signal at the boundary compared to intra-tissue contacts. A significant decrease was observed for ΔCEph, eB1 MO, and dominant negative Rac (* *p*<0.05; ** *p*<0.01).
(2.89 MB TIF)

Text S1 Supplementary methods. Plasmids, mRNAs, and antisense oligonucleotides used for injection.
(0.05 MB DOC)

Movie S1 Wild type mesoderm on BCR (ectoderm). Cells within the tissue move together and are tightly apposed. Adjacent cells at the boundary continuously make and break contacts. At sites of contacts across the boundary, cells move in concert indicating that cells have established stable adhesion. At sites of retraction, membranes of the mesoderm cells show wrinkles. Retraction fibers are observed during detachment. Asterisk indicates a gap within the mesoderm explants. Note that cells do not detach, but instead close the gap, showing protrusive activity.
(5.79 MB MOV)

Movie S2 Ectoderm on BCR (ectoderm). Cells move coherently both within the explant and at the interface of the explant and substrate. Adjacent cells at the interface immediately establish contacts which are never disrupted.
(8.26 MB MOV)

Movie S3 Control MO-injected mesoderm on wild type BCR. Mesoderm cell retracts, leaving behind retraction fibers. Cell from the left hand side glides past BCR cells while extending protrusions, then retracts, then glides again.
(0.27 MB MOV)

Movie S4 EphrinB2MO-injected mesoderm on wild type BCR. eB2 MO-injected mesoderm cells fail to retract. They move co-ordinately with adjacent BCR cells and mix with them, indicating establishment of stable contacts with the BCR, which are maintained throughout the duration of the movie.
(0.49 MB MOV)

Movie S5 Wild type mesoderm on ephrinB1 MO BCR. Wild type mesoderm cells fail to retract from ephrinB1 MO injected ectoderm. They move co-ordinately with morphant BCR cells and mix with them, indicating establishment of stable contacts, which are maintained throughout the duration of the movie.
(0.28 MB MOV)

Movie S6 Ectoderm on ectoderm. Control for Movies S3–S5.
(1.17 MB MOV)

Movie S7 V14RhoA co-expressed with ΔCEphMO rescues separation. Inhibition of repulsion by ΔCEph in the BCR is rescued by co-expression of constitutively active V14RhoA. Spots mark the two parallel but separated membranes of cells that remain detached for more than 6 frames and slide past each other at the boundary. The movie is paused for two time frames where the spots appear.
(0.89 MB MOV)

Movie S8 V12Rac co-expressed with ΔCEphMO rescues separation. Inhibition of repulsion by ΔCEph in the BCR is rescued by co-expression of constitutively active V12Rac. Arrows indicate the points where the three mesoderm cells are attached to the ectoderm. The two cells on the right start to detach first (arrowheads), the third cell follows later. Spots mark the membrane of this cell that remains detached until the end of the movie. The movie is paused for two time frames where the spots appear.
(0.45 MB MOV)

Movie S9 GFP-Rthk-GBD. The upper mesoderm cell establishes contact with a BCR cell, then retracts. GFP-Rhothekin-GBD concentrates at the site of contact and dissipates after detachment. The lower cell accumulates progressively higher levels of GFP-Rhothekin-GBD but fails to retract. Time between frames: 2 min.
(0.74 MB MOV)

Movie S10 GFP-Wasp-GBD. Two cells are initially in close contact with the BCR. The upper cell remains in contact throughout this sequence, while the lower cell detaches. The intense GFP-Wasp-GBD signal at the sites of contact dissipates slowly in both cells. Time between frames: 1 min.
(0.37 MB MOV)

## Abbreviations

BCR: blastocoel roof
GBD: GTPase-binding domain
MO: morpholino antisense oligonucleotide
PAPC: paraxial protocadherin
