# Supplementary figures and images for: EphrinB/EphB Signaling Controls Embryonic Germ Layer Separation by Contact-Induced Cell Detachment
Source: PLoS Biol. 2011 Mar 1;9(3):e1000597. doi: 10.1371/journal.pbio.1000597 (PMC3046958; doi:10.1371/journal.pbio.1000597)

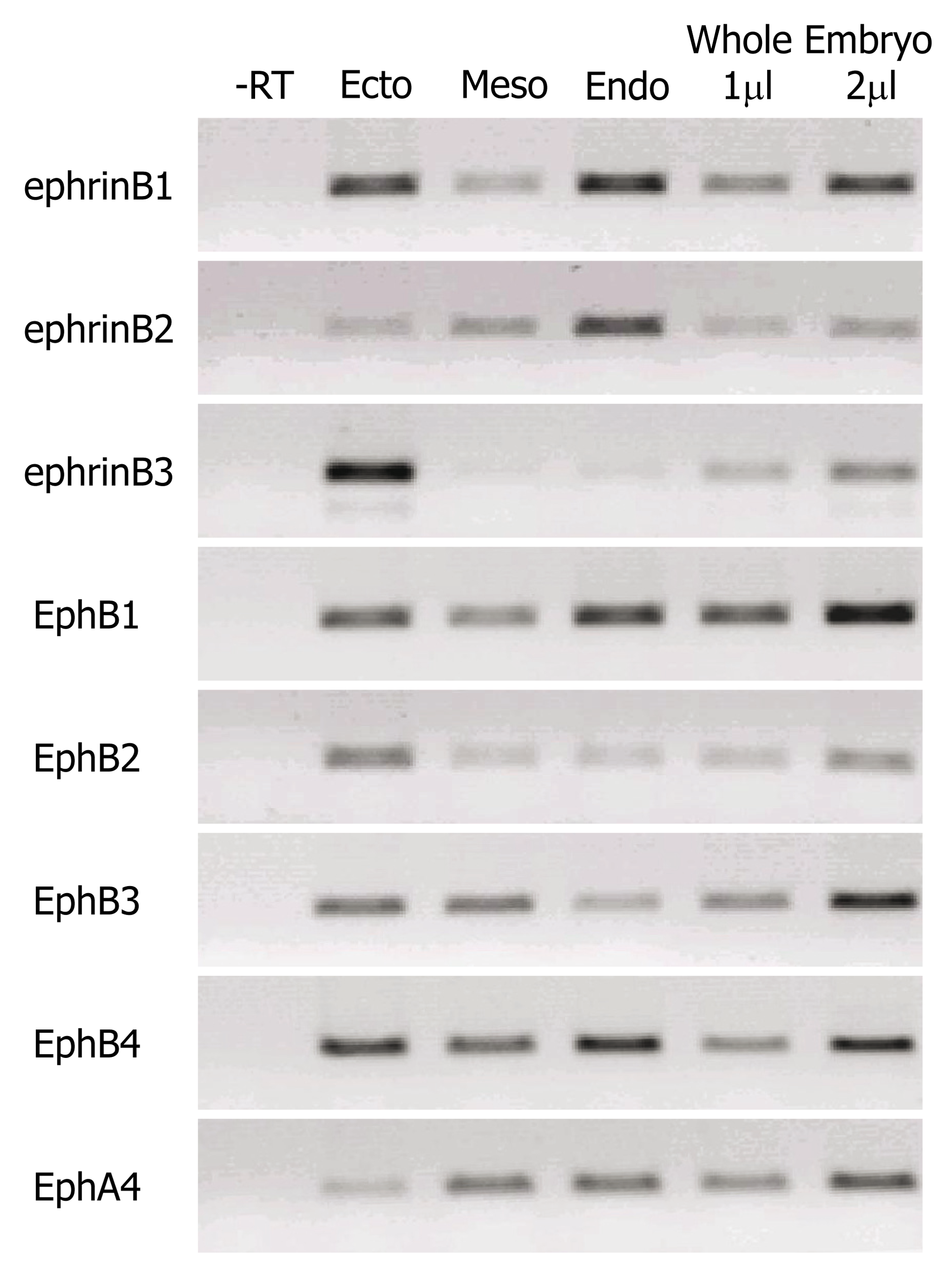

Supplement: Figure S1 — Expression ephrinBs and EphBs in the three germ layers at early gastrula stage. RT-PCR was performed using mRNA extracted from ectoderm, dorsal mesoderm, and endoderm tissues dissected at stage 10.5. Loading was equalized by comparing levels of FGFR in the three tissues (unpublished data). Two independent experiments showed identical patterns of expression. (2.25 MB TIF) [file pbio.1000597.s001.tif]

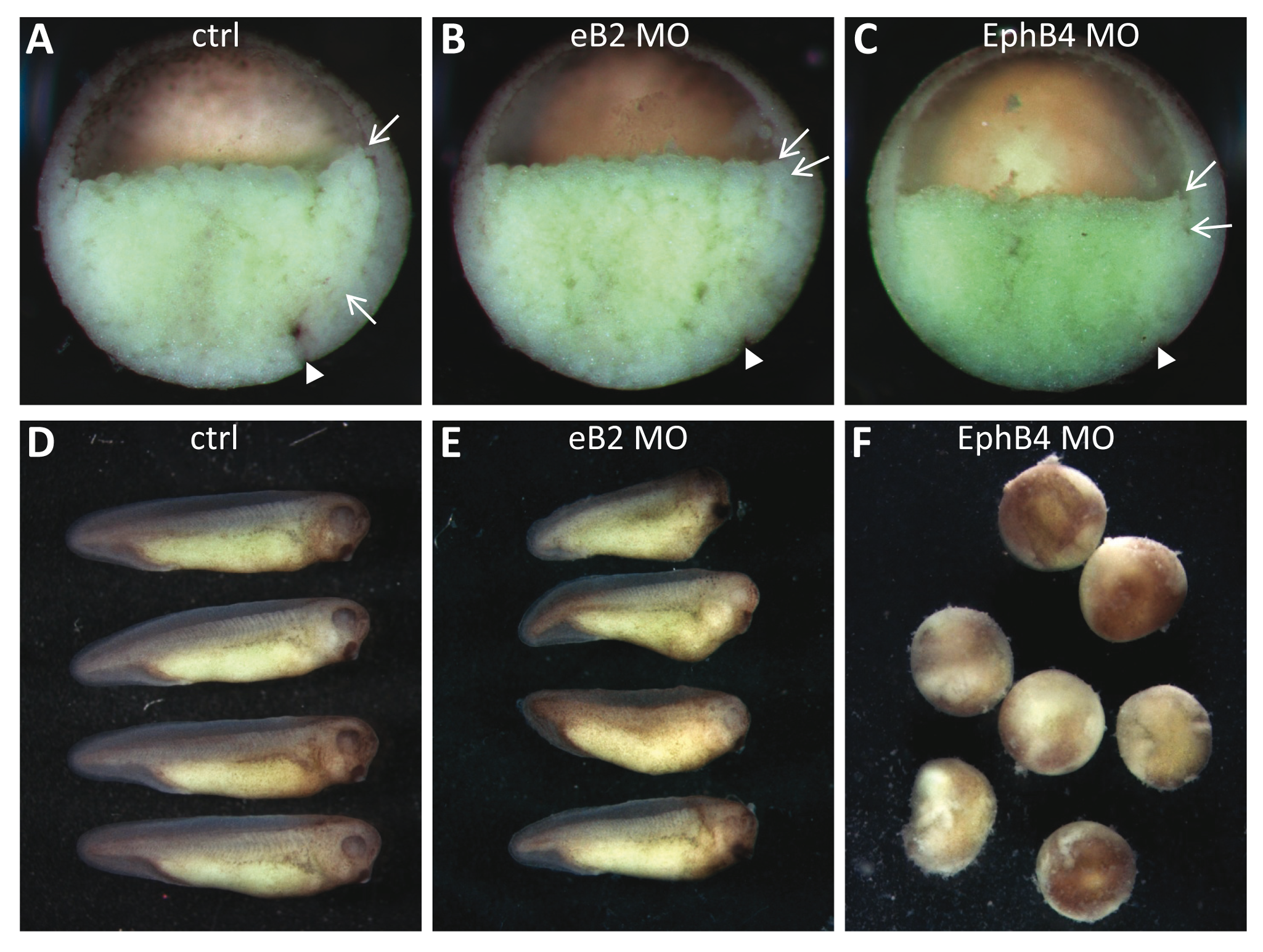

Supplement: Figure S2 — Whole embryo phenotypes for ephrinB2 and EphB4 depletion. EphrinB2 (eB2) and EphB4 MOs were injected in the two blastomeres of the two-cell stage embryo. Embryos were fixed at the early gastrula stage and bisected sagitally (A–C) or allowed to develop until early tadpole stages (D–F). (A–C) Arrows point to both ends of Brachet's cleft. (3.38 MB TIF) [file pbio.1000597.s002.tif]

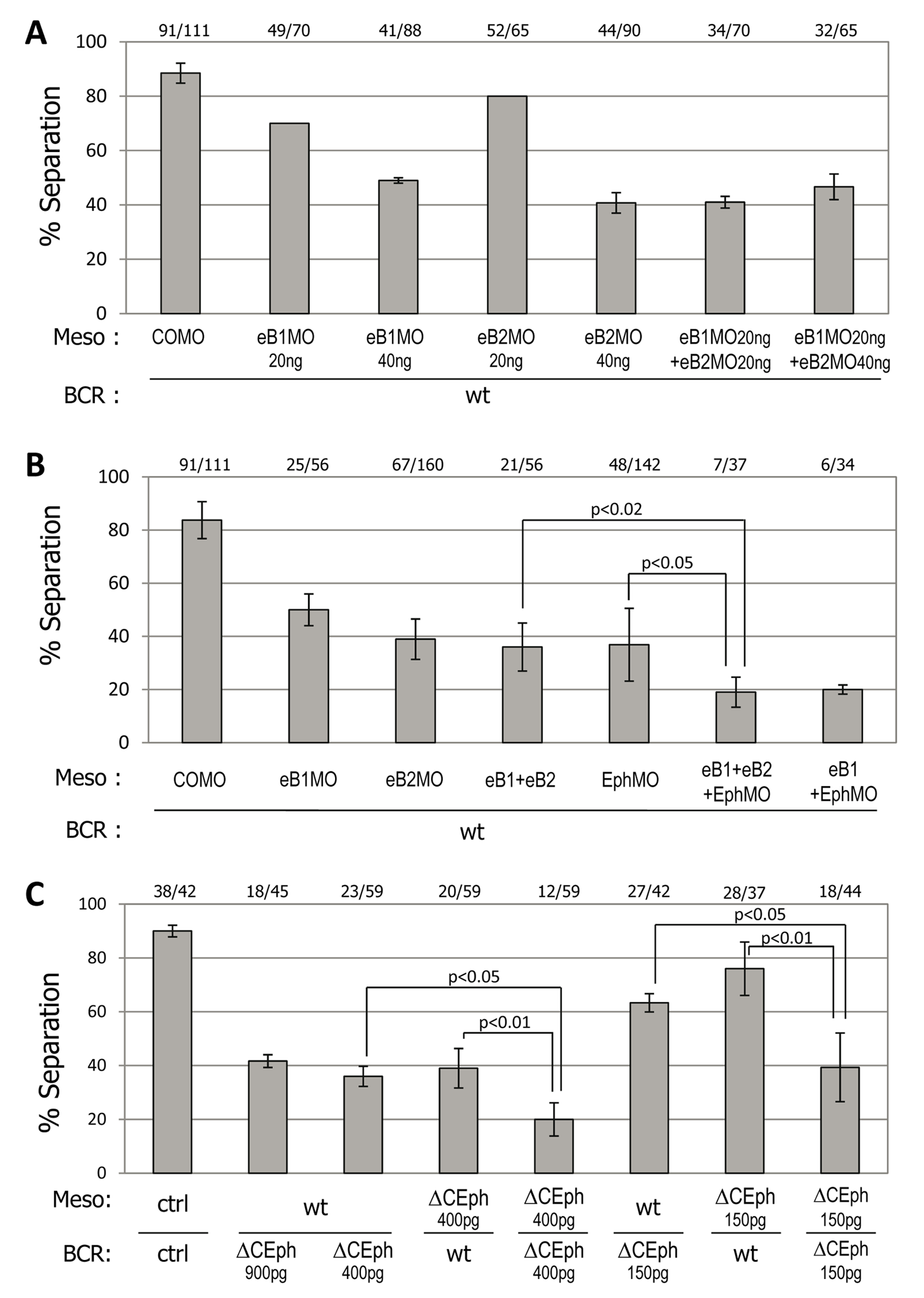

Supplement: Figure S3 — Multiple interference with ephrinB1, B2, and EphB. (A) Single and double EphrinB1 and B2 knockdown in the mesoderm by injection of various amounts of eB1 and eB2 MOs. Single MOs caused significant separation, demonstrating that both ephrins are required. Inhibition was dose dependent. It was not increased by co-injection of eB1 MO and eB2 MO, even with the highest amounts of MO, indicating that mesodermal ephrins contribute only partly to separation. (B) Simultaneous injection of EphB4 MO with eB2 MO or eB1+eB2 MO caused stronger inhibition of separation compared to single EphB4, eB2, or eB1+eb2 MO injections. (C) EphB inhibition by expression of dominant negative ΔC-EphB. EphB activity is required in both ectoderm and mesoderm. Inhibition by ΔC-EphB was dose dependent but reached a maximum at 400 pg. A significantly stronger effect was obtained by simultaneous interference in both tissues, both with levels yielding maximal (400 pg) or submaximal inhibition (150 pg). (0.80 MB TIF) [file pbio.1000597.s003.tif]

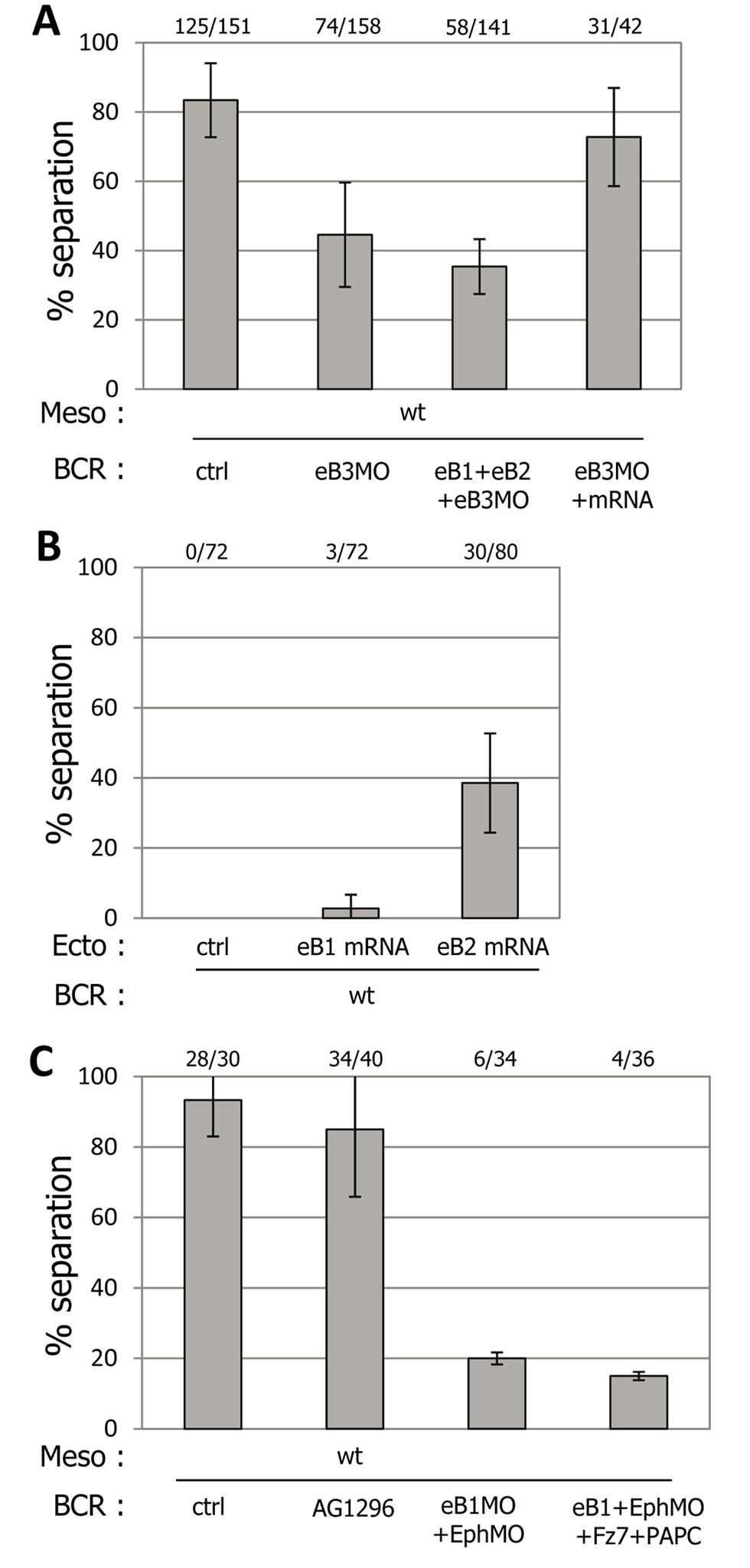

Supplement: Figure S4 — (A) Interference with ephrin B3 in the BCR. Injection of eB3 MO (40 ng) in the BCR caused inhibition of separation (p = 7.40E-07). The degree of inhibition upon triple injection of eB1, eB2, and eB3 MO was similar to eB1 MO alone (see Figure 2C). Separation was rescued by co-injection of eB3 mRNA. (B) EphrinB2 overexpression is sufficient to induce separation behavior in the ectoderm. Embryos were injected with ephrinB1 or ephrinB2 mRNA (500 pg/injection). EphrinB2 induced separation (p = 0.01) while ephrinB1 had no effect (p = 0.24). (C) Effect of PDGF and Fz/PAPC signaling. Treatment of mesoderm explants with PDGF receptor kinase inhibitor AG1296 (10 µM) does not inhibit separation. Expression of Fz7 and PAPC does not rescue separation when co-injected with eB1 and EphB4 MOs. (0.43 MB TIF) [file pbio.1000597.s004.tif]

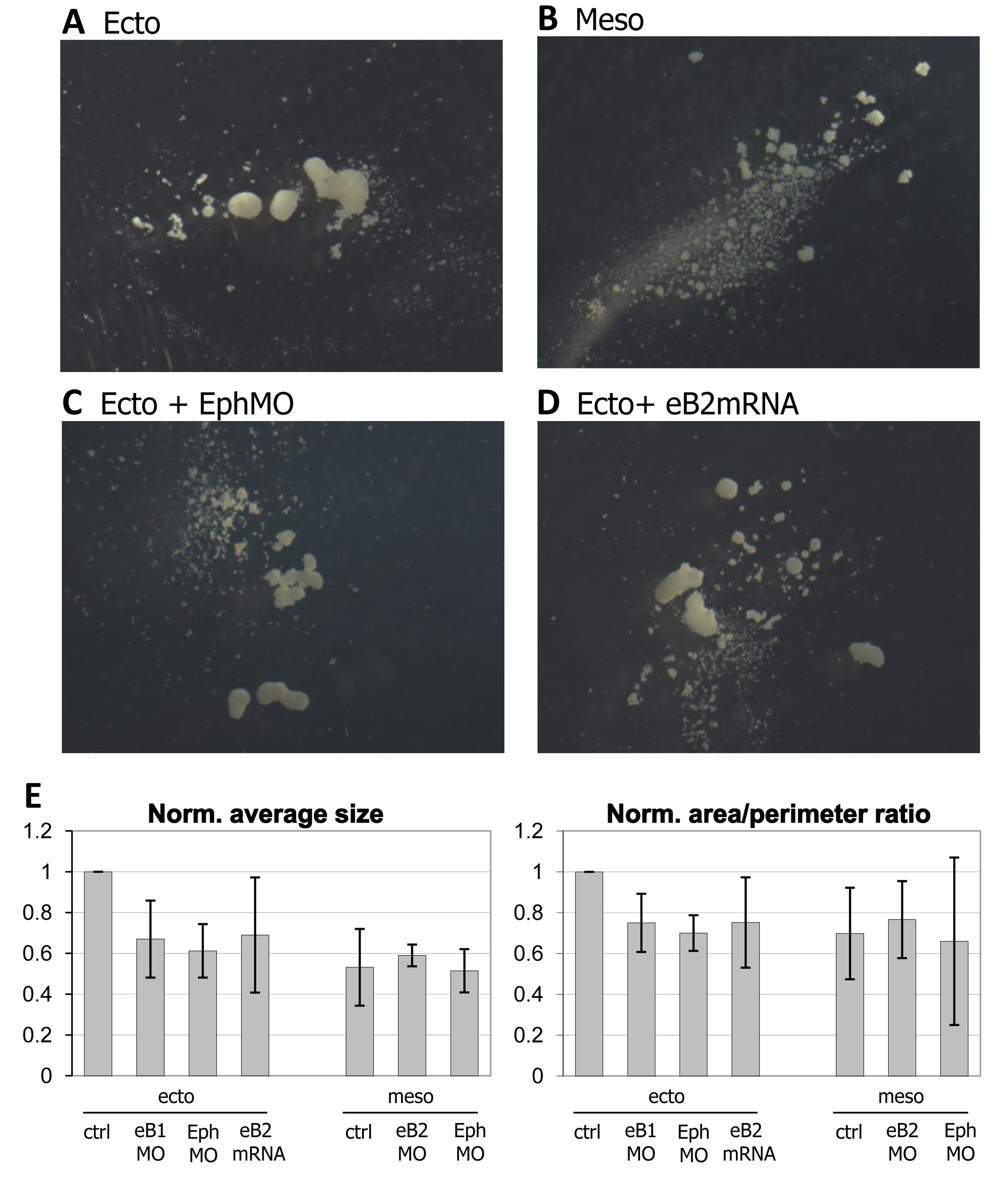

Supplement: Figure S5 — Reaggregation. Dissociated ectoderm and mesoderm cells were left to reaggregate for 1 h. (A–D) Reaggregated wild type ectoderm (A), wild type mesoderm (B), ephrinB1-depleted ectoderm (C), and ephrinB2 overexpressing ectoderm (D). (E) The degree of reaggregation was determined using two criteria: the average particle size, reflecting the extent of aggregation, and the total area/perimeter ratio, which integrates both the size of the aggregates and their degree of compaction (single cells and small aggregates have a large area/perimeter ratio, large round aggregates have a minimal perimeter, thus a higher area/perimeter ratio). Results from individual experiments were normalized using wild type ectoderm as reference (1.0) to account for batch-to-batch variation. Both parameters gave similar results, and the same trend for each of the condition was observed at earlier time points (unpublished data): mesoderm reaggregated less rapidly than ectoderm. EphrinB1 and EphB4 depletions decreased ectoderm reaggregation. EphrinB2 overexpression lead to a similar, although more variable, inhibition. EphrinB2 and EphB4 depletion had no effect in the mesoderm. (1.90 MB TIF) [file pbio.1000597.s005.tif]

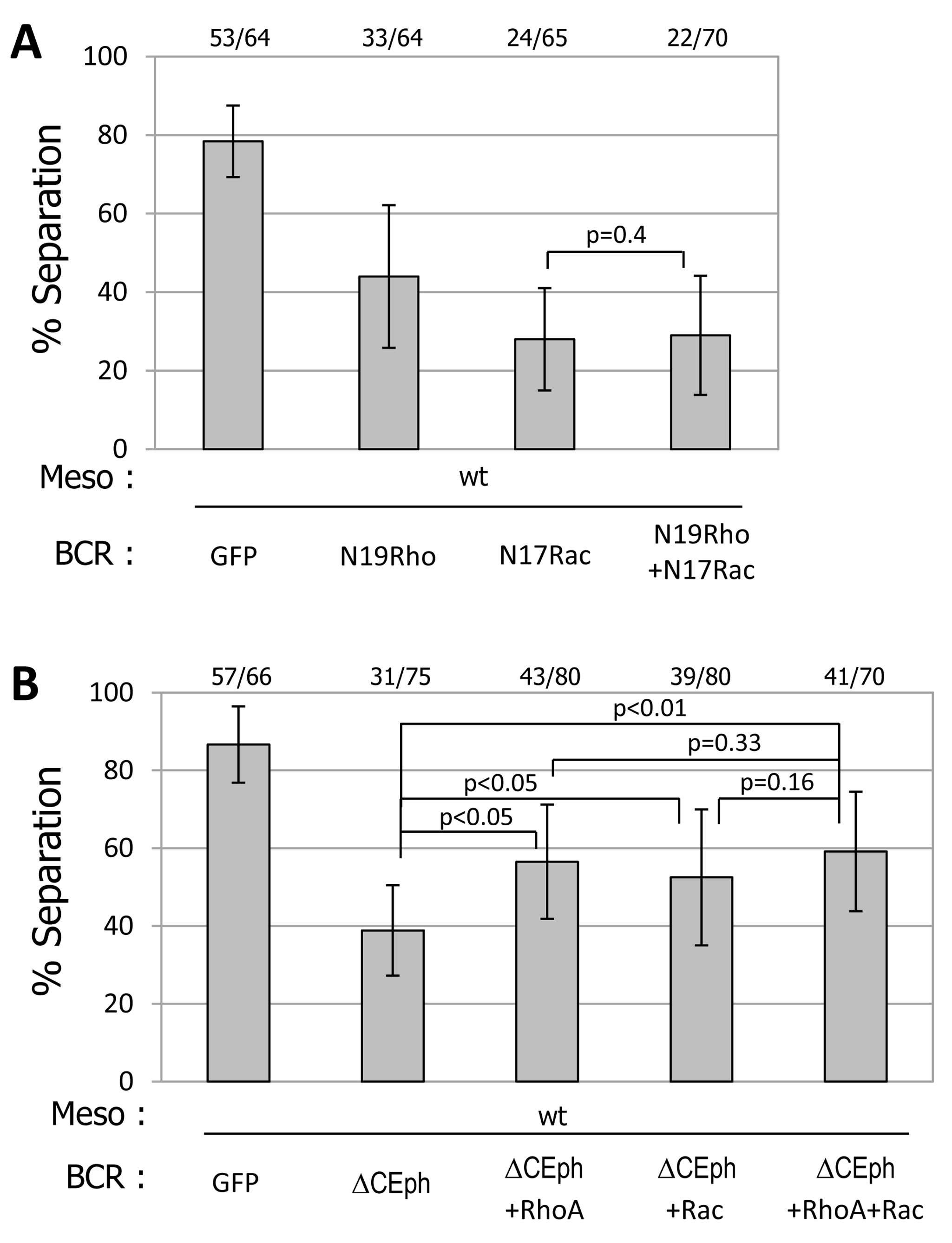

Supplement: Figure S6 — Combined RhoA/Rac interference and rescue with wild type RhoA and Rac. (A) Dominant negative N19RhoA and N17Rac (100 pg mRNA) were expressed alone or in combination in the BCR. Double inhibition of RhoA and Rac did not significantly enhance inhibition of separation (data from six independent experiments). (B) ΔCEph was expressed alone or with wild type RhoA or Rac. Inhibition of separation by ΔCEph is weakly rescued by wild type RhoA and Rac. Rescue was not enhanced by simultaneous expression of both RhoA and Rac. Data were pooled from three experiments with doses of 100 pg and three experiments with doses of 200 pg RhoA or Rac mRNA. The strength of rescue was similar at both doses. (0.86 MB TIF) [file pbio.1000597.s006.tif]

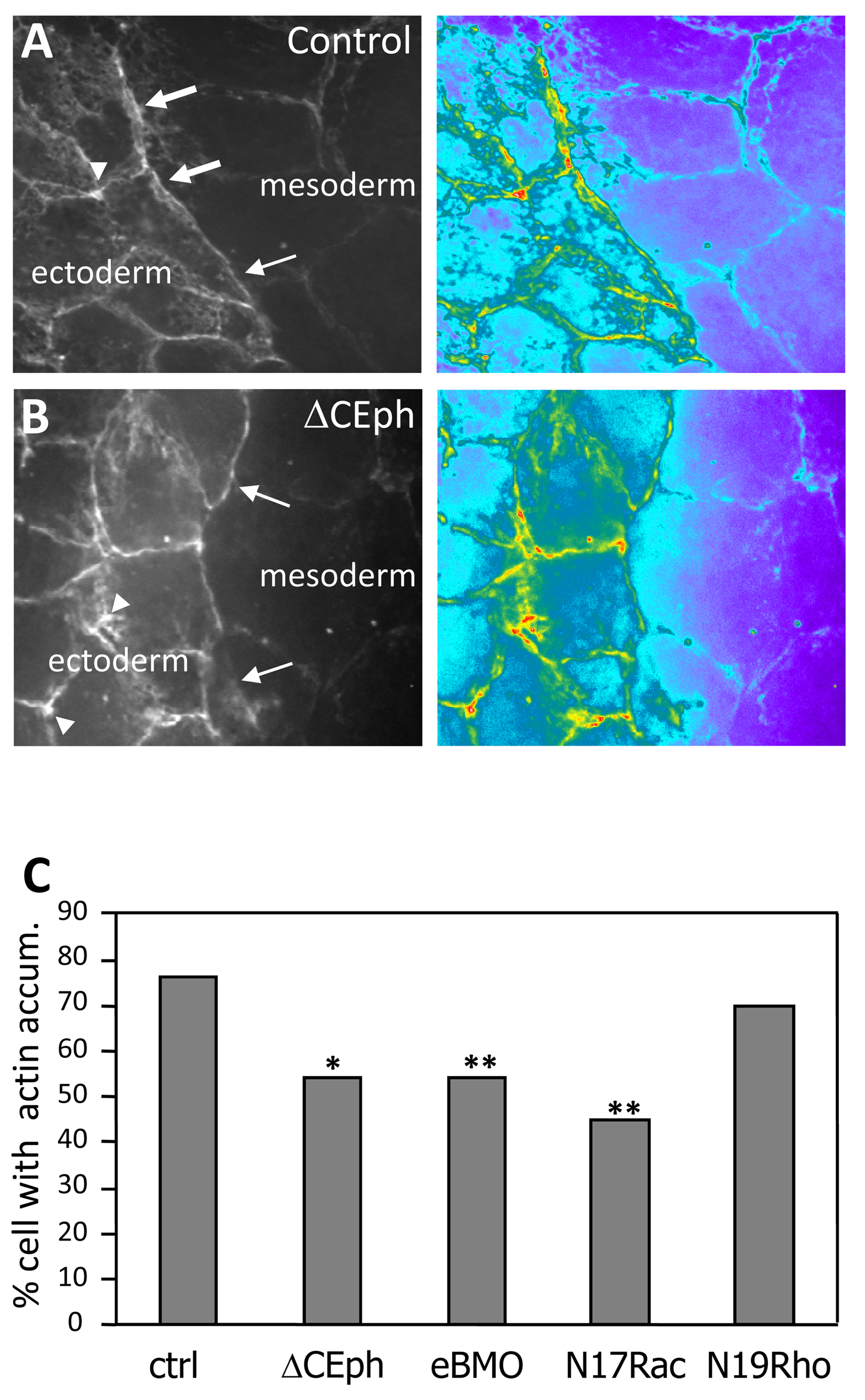

Supplement: Figure S7 — Eph-dependent F-actin accumulation at the mesoderm/BCR boundary. Mesoderm explants were combined with BCRs manipulated by injection of ΔCEph, N17Rac, N19RhoA mRNAs, or eB1 MO. Sample were fixed and stained with Alexa488-phalloidin and analyzed by confocal microscopy. Stacks of five focal planes (2 µm) were merged, and relative intensity levels were compared using pseudocolors. (A) Boundary between mesoderm and BCR. F-actin is concentrated at cell cortex, with BCR cells having a stronger signal than mesoderm cells. The boundary (arrows) showed F-actin accumulations (thick arrows) similar to those found at some tri-cellular junctions in the BCR (arrowheads). (B) Mesoderm combined with ΔCEph-expressing ectoderm. F-actin accumulation at the mesoderm/BCR interface (arrows) was weaker than in controls. (C) Quantitation of F-actin accumulation at the boundary. Using pseudocolors, BCR cells were scored for stronger signal at the boundary compared to intra-tissue contacts. A significant decrease was observed for ΔCEph, eB1 MO, and dominant negative Rac (* p<0.05; ** p<0.01). (2.89 MB TIF) [file pbio.1000597.s007.tif]
