# Supplementary material for: EphrinB/EphB Signaling Controls Embryonic Germ Layer Separation by Contact-Induced Cell Detachment
Source: PLoS Biol. 2011 Mar 1;9(3):e1000597. doi: 10.1371/journal.pbio.1000597 (PMC3046958; doi:10.1371/journal.pbio.1000597)
Supplement: Text S1 — Supplementary methods. Plasmids, mRNAs, and antisense oligonucleotides used for injection. (0.05 MB DOC) [file pbio.1000597.s008.doc]

**Supplementary material and methods**

**Morpholinos:**

| Target | Sequence |
| --- | --- |
| ephrinB1 | GGAGCCCTTCCATCCGCACAGGTGG |
| ephrinB2 | ACACCGAGTCCCCGCTCAGTGCCAT |
| ephrinB2a | ACACCGAGTCCCCGCTCAGTGCCAT |
| ephrinB2b | ACACCGAGTCCCCGCTCAGTGCCAT |
| EphB4a | ACAGGAGGAGGAGCCAGAGATCCAT |
| control | CCTCTTACCTCAGTTACAATTTATA |

| Amounts injected per blastomere | |
| --- | --- |
| ephrinB1 | 40ng |
| ephrinB2 | 40ng |
| ephrinB2a | 40ng |
| ephrinB2b | 40ng |
| EphB4a | 30ng |
| control | 40ng |
| ephrinB1 + ephrinB2 | 20ng+20ng |
| ephrinB2a +ephrinB2b | 20ng+20ng |
| ephrinB1 + ephrinB2 + EphB4 | 20ng+20ng  +30ng |

**mRNAs:**

| mRNA amounts injected per blastomere: | |
| --- | --- |
| EphrinB1 | 250pg |
| EphrinB1 delta c | 250pg |
| EphrinB2 | 50pg |
| EphB4 | 250pg |
| EPhB4 delta c | 400pg |
| caRac | 12.5-25pg |
| caRho | 12.5-25pg |
| GFP | 25-100pg |
| Bgal | 100pg |
| Fz7 | 400pg |
| PAPC | 500pg |

**Plasmids**

Wild type and delta-cytoplasmic (1-268) ephrinB1 in pSP64TEN were gifts of Dr. Ira Daar [1].Full length ephrinB2 was PCR amplified from clone BC057724 (Open Biosystem) and inserted in Stu1/Xba1 sites of pCS2+. Wild type and delta-cytoplasmic EphB4 in pCS2+ were gifts from Dr. A. Brändli [2]. XN19RhoA and XV14RhoA in pBS [3], and hN17Rac1 and hV12Rac1 in pCS2+ [4] were gifts of Dr. Ken Cho (Univ. of california) and Dr. K. Symes (Univ. of Boston). Myc-tagged GFP and β-galactosidase are in pCS2+ [5,6]. GAP43-GFP (mGFP) in pCS2+ was a gift of Dr. Eddy DeRobertis (UCLA). mYFP and mCherry were constructed by inserting an oligonucleotide corresponding to the region coding for the first 20 amino acids of GAP43 upstream eGPF/eYFP into the EcoR1 site of pCS2. xFrizzled 7 in pCS2+ [7] was a gift of Dr. Steinbeisser (Univ. of Heidelberg). xPAPC in pCMV-SPORT6 was obtained from Open Biosystems (clone ID # 5542127) and recloned into pCS2+.

**References**

1. Jones TL, Chong LD, Kim J, Xu R-H, Kung H-F, et al. (1998) Loss of cell adhesion in Xenopus laevis embryos mediated by the cytoplasmic domain of XLerk, an erythropoietin-producing hepatocellular ligand. PNAS 95: 576-581.

2. Helbling PM, Saulnier DME, Robinson V, Christiansen JH, Wilkinson DG, Brändli A (1999) Comparative analysis of embryonic gene expression defines potential interaction sites for Xenopus EphB4 receptors with ephrin-B ligands. Dev Dyn 216: 361-373.

3. Wunnenberg-Stapleton K, Blitz IL, Hashimoto C, Cho KW (1999) Involvement of the small GTPases XRhoA and XRnd1 in cell adhesion and head formation in early Xenopus development. Development 126: 5339-5351.

4. Tahinci E, Symes K (2003) Distinct functions of Rho and Rac are required for convergent extension during Xenopus gastrulation. Dev Biol 259: 318-335.

5. Reintsch WE, Habring-Müller A, Wang R, Schohl, A Fagotto F (2005) β-catenin controls cell sorting at the notochord–somite boundary independently of cadherin-mediated adhesion. J Cell Biol 170: 675-686.

6. Rupp RA, Snider L, Weintraub H (1994) [Xenopus embryos regulate the nuclear localization of XMyoD.](http://www.ncbi.nlm.nih.gov/pubmed/7926732) Genes Dev 8: 1311-1323.

7. Medina A, Steinbeisser H (2000) Interaction of Frizzled 7 and Dishevelled in *Xenopus*. Dev Dyn 218: 671-680.
